# TAO-DFT-Based *Ab Initio* Molecular Dynamics

**DOI:** 10.3389/fchem.2020.589432

**Published:** 2020-11-05

**Authors:** Shaozhi Li, Jeng-Da Chai

**Affiliations:** ^1^Department of Physics, National Taiwan University, Taipei, Taiwan; ^2^Center for Theoretical Physics and Center for Quantum Science and Engineering, National Taiwan University, Taipei, Taiwan

**Keywords:** TAO-DFT, AIMD, static correlation, radical nature, infrared spectra

## Abstract

Recently, AIMD (*ab initio* molecular dynamics) has been extensively employed to explore the dynamical information of electronic systems. However, it remains extremely challenging to reliably predict the properties of nanosystems with a radical nature using conventional electronic structure methods (e.g., Kohn-Sham density functional theory) due to the presence of static correlation. To address this challenge, we combine the recently formulated TAO-DFT (thermally-assisted-occupation density functional theory) with AIMD. The resulting TAO-AIMD method is employed to investigate the instantaneous/average radical nature and infrared spectra of *n*-acenes containing *n* linearly fused benzene rings (*n* = 2–8) at 300 K. According to the TAO-AIMD simulations, on average, the smaller *n*-acenes (up to *n* = 5) possess a nonradical nature, and the larger *n*-acenes (*n* = 6–8) possess an increasing radical nature, showing remarkable similarities to the ground-state counterparts at 0 K. Besides, the infrared spectra of *n*-acenes obtained with the TAO-AIMD simulations are in qualitative agreement with the existing experimental data.

## 1. Introduction

Molecular dynamics (MD) is a computational method for simulating dynamical processes that occur in a system consisting of atoms (for example, atoms, molecules, solids, and liquids) (Lifson and Warshel, 1968; Levitt and Lifson, 1969; Karplus and Petsko, 1990; Kresse and Hafner, 1993; Sprik et al., 1996; Silvestrelli et al., 1997; Putrino and Parrinello, 2002; Tuckerman, 2002; Chai et al., 2003; Kuo and Mundy, 2004; Jensen, 2007; Marx and Hutter, 2009; Gaigeot, 2010; Ramírez-Solís et al., 2011; Vitale et al., 2015; Hollingsworth et al., 2018). The motion of the atomic nuclei in the system is described by the classical Newtonian equations of motion, starting from pre-specified initial conditions (e.g., initial nuclear positions and velocities) and subject to boundary conditions suitable for the system studied. By performing MD simulations, one can calculate both the dynamical and equilibrium thermodynamic properties associated with a system at non-zero temperatures and can simultaneously monitor the microscopic movements of the atomic nuclei in the system. However, the specification of the forces acting on the atomic nuclei in MD simulations remains challenging, yielding two popular types of MD methods: classical MD and *ab initio* MD (AIMD).

In classical MD, the forces that act on the atomic nuclei are calculated using an empirical potential energy function (i.e., the potential energy of a system is expressed as a function of the nuclear coordinates) that is defined by molecular mechanics (Tuckerman, 2002; Jensen, 2007; Marx and Hutter, 2009). Because of a simple analytical function for the potential energy, classical MD is computationally very efficient, and it has thus been widely employed for many applications (Lifson and Warshel, 1968; Levitt and Lifson, 1969; Karplus and Petsko, 1990; Hollingsworth et al., 2018). For example, classical MD can reveal the details of the motion of macromolecules (e.g., proteins) (Lifson and Warshel, 1968; Levitt and Lifson, 1969), which can be very challenging using experimental approaches. However, in classical MD, empirical potential energy functions are typically parameterized for some peculiar systems and may not be transferable to others (Tuckerman, 2002). Furthermore, in classical MD, electrons are not present explicitly (i.e., their effects are approximated by empirical potential energy functions), and the electronic properties (e.g., electron density and radical nature) of systems therefore cannot be explored.

To overcome the shortcomings of classical MD, one can resort to AIMD, wherein the forces that act on the atomic nuclei are calculated on-the-fly according to the potential energy obtained with an electronic structure method along an AIMD trajectory (Tuckerman, 2002; Jensen, 2007; Marx and Hutter, 2009). Clearly, the accuracy of AIMD simulations can be limited due to the accuracy of the underlying electronic structure method. On the other hand, for AIMD simulations, it is also essential to adopt a computationally efficient electronic structure method that can provide reasonably accurate potential energy for the systems of interest, since there are typically 10^4^–10^6^ time steps per AIMD trajectory. Owing to the decent balance between performance and cost, KS-DFT (i.e., Kohn-Sham density functional theory) (Kohn and Sham, 1965) is currently the most popular electronic structure method adopted in AIMD simulations. Over the past three decades, KS-DFT-based AIMD (KS-AIMD) has been commonly used to investigate the equilibrium thermodynamic and dynamical properties associated with electronic systems at finite temperatures, and has been a powerful AIMD method for modeling various phenomena (Kresse and Hafner, 1993; Sprik et al., 1996; Silvestrelli et al., 1997; Putrino and Parrinello, 2002; Chai et al., 2003; Kuo and Mundy, 2004; Gaigeot, 2010; Ramírez-Solís et al., 2011; Vitale et al., 2015).

**Figure 1:**
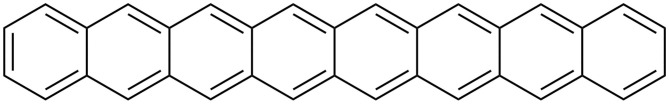
Structure of 8-acene, containing eight linearly fused benzene rings.

Nevertheless, KS-DFT using traditional exchange-correlation (XC) energy functionals could yield qualitative failures for the various properties associated with electronic systems possessing radical nature due to the lack of static correlation effects (Cohen et al., 2008, 2012). For example, consider linear *n*-acene (with the chemical formula C_4*n*+2_H_2*n*+4_), which contains *n* linearly fused benzene rings (for example, see Figure 1), *N*_*e*_ = 26*n* + 16 electrons, and *N* = 6*n*+6 nuclei. According to the recent findings (Hachmann et al., 2007; Chai, 2012, 2014, 2017; Rivero et al., 2013; Wu and Chai, 2015; Fosso-Tande et al., 2016), the larger *n*-acenes (*n* ≥ 6) possess increasing radical nature in their ground states. However, KS-DFT adopting commonly used XC energy functionals could perform poorly for the larger *n*-acenes (*n* ≥ 6) due to the lack of static correlation effects (Hachmann et al., 2007; Chai, 2012, 2017). It can therefore be anticipated that KS-AIMD simulations at finite temperatures may yield unreliable dynamical information for electronic systems with a radical nature [e.g., the larger *n*-acenes (*n* ≥ 6)].

To study the properties of electronic systems with a radical nature, one generally resorts to multi-reference (MR) electronic structure methods (Andersson et al., 1992; Hachmann et al., 2007; Gidofalvi and Mazziotti, 2008; Pelzer et al., 2011; Gryn'ova et al., 2015; Fosso-Tande et al., 2016; Battaglia et al., 2017; Mullinax et al., 2019). Although MR electronic structure methods can reliably predict the various properties associated with electronic systems with radical nature, they could be prohibitively expensive for a single-point energy + nuclear gradient calculation on large electronic systems, not to mention the respective AIMD simulations where such calculations should be performed about 10^4^–10^6^ times per AIMD trajectory. To explore the dynamical properties of nanosystems with radical nature using AIMD simulations at finite temperatures, therefore, it is essential to employ an efficient electronic structure method that can properly describe the static correlation effects during the AIMD simulations.

Recently, TAO-DFT (thermally-assisted-occupation density functional theory) (Chai, 2012) has been formulated for investigating the ground-state (GS) properties associated with nanosystems possessing a radical nature at 0 K. Note that TAO-DFT is a density functional theory adopting fractional orbital occupation numbers (i.e., generated by the Fermi-Dirac distribution using a fictitious temperature θ), which is rather different from KS-DFT. In TAO-DFT, the fictitious temperature θ is intimately correlated with the effect of the configuration mixing on the electron density of a GS system (e.g., see sections 2, 3 of Chai, 2012), and it is thus completely unrelated to the physical temperature (i.e., 0 K) of the GS system. Note that KS-DFT corresponds to TAO-DFT with θ = 0. However, for a general GS system at 0 K, the θ value in TAO-DFT can be nonzero (e.g., see sections 3.5, 4 of Chai, 2012). In TAO-DFT, an entropy contribution term, which is dependent on the fictitious temperature θ and orbital occupation numbers, can offer a proper description for static correlation even when the simplest LDA (i.e., local density approximation) XC energy functional is used (Chai, 2012). At the LDA level, even with a properly defined system-independent θ value (e.g., see section 5 of Chai, 2012), TAO-DFT, which has similar cost as KS-DFT in computation, has been shown to consistently outperform KS-DFT for electronic systems with a radical nature, while performing similarly to KS-DFT for electronic systems with a nonradical nature (Chai, 2012).

More complicated semilocal (Chai, 2014), global hybrid (Chai, 2017), and range-separated hybrid (Chai, 2017; Xuan et al., 2019) XC energy functionals could also be used in TAO-DFT. Moreover, in order to enhance its accuracy for a wide range of applications, a method that determines the θ value in TAO-DFT in a self-consistent manner has recently been developed (Lin et al., 2017). Very recently, a frequency-domain formulation of linear-response time-dependent TAO-DFT (Yeh et al., 2020) has been formulated to explore the properties of electronic excited states within TAO-DFT.

Owing to its computational efficiency and decent accuracy for exploring the properties of electronic systems at the nanoscale, TAO-DFT has recently been adopted to investigate the electronic properties (Wu and Chai, 2015; Seenithurai and Chai, 2016, 2017, 2018, 2019, 2020; Wu et al., 2016; Yeh and Chai, 2016; Yeh et al., 2018; Chung and Chai, 2019; Deng and Chai, 2019; Hanson-Heine and Hirst, 2020; Hanson-Heine et al., 2020; Huang et al., 2020; Manassir and Pakiari, 2020), hydrogen storage properties (Seenithurai and Chai, 2016, 2017, 2018), and vibrational frequencies (Hanson-Heine, 2020) of several electronic systems at the nanoscale, especially for those possessing a radical nature.

Therefore, in the present work, we propose to combine TAO-DFT (Chai, 2012) with AIMD, yielding TAO-DFT-based AIMD (TAO-AIMD). Since analytical nuclear gradients for TAO-DFT are available (Chai, 2012), TAO-AIMD is as computationally efficient as KS-AIMD. Accordingly, it is feasible to study the equilibrium thermodynamic and dynamical properties of nanosystems with a radical nature using TAO-AIMD simulations at finite temperatures. To highlight some of the present capabilities of TAO-AIMD, we perform TAO-AIMD simulations to explore the instantaneous/average radical nature and infrared (IR) spectra of *n*-acenes with *n* = 2–8 fused benzene rings at 300 K. The rest of this paper is organized as follows. The TAO-AIMD method is defined in section 2. We then describe the computational details in section 3, discuss the results in section 4, and give our conclusions in section 5.

## 2. TAO-AIMD

Consider a system containing *N*_*e*_ electrons (described by coordinates **r**_1_, …, **r**_*N*_*e*__) and *N* nuclei (described by coordinates **R**_1_, …, **R**_*N*_). Here, we resort to the adiabatic or Born-Oppenheimer (BO) approximation (Born and Oppenheimer, 1927). Because the electrons are much lighter than the nuclei, it is assumed that the nuclei move relatively slowly, and hence the electrons are able to respond to the nuclear motion almost instantaneously. In other words, the electronic motion and nuclear motion can be treated separately.

Accordingly, in the first step of the BO approximation, the kinetic energy of the nuclei is ignored. For fixed nuclear positions **R**_1_, …, **R**_*N*_, the electronic Hamiltonian is expressed as (Tuckerman, 2002; Jensen, 2007; Marx and Hutter, 2009)

(1) $$\begin{array}{l} {Ĥ}_{\text{elec}}=-\frac{\hbar^{2}}{2m_{e}}\overset{N_{e}}{\underset{i=1}{\sum }}\nabla_{i}^{2}-\frac{e^{2}}{4\pi \epsilon_{0}}\overset{N_{e}}{\underset{i=1}{\sum }}\overset{N}{\underset{A=1}{\sum }}\frac{Z_{A}}{\left|{\text{r}}_{i}-{\text{R}}_{A}\right|}\\ \;+\frac{e^{2}}{4\pi \epsilon_{0}}\overset{N_{e}}{\underset{i=1}{\sum }}\overset{N_{e}}{\underset{j>i}{\sum }}\frac{1}{\left|{\text{r}}_{i}-{\text{r}}_{j}\right|}, \end{array}$$

where *m*_*e*_ and −*e* are the mass and charge, respectively, of an electron, and *Z*_*A*_*e* is the charge of nucleus *A*. On the right-hand side of Equation (1), the first term is the kinetic energy of electrons, the second term is the nuclear-electron attraction energy, and the third term is the electron-electron repulsion energy. The time-independent electronic Schrödinger equation

(2) $$\begin{array}{l} {Ĥ}_{\text{elec}}{\text{Ψ}}_{k}=E_{k}{\text{Ψ}}_{k} \end{array}$$

is subsequently solved for the electronic energy *E*_*k*_ and electronic wavefunction Ψ_*k*_. In particular, the lowest eigenvalue *E*_0_ is the GS electronic energy, and the corresponding eigenfunction Ψ_0_ is the GS electronic wavefunction. By adding the nuclear-nuclear repulsion energy to the electronic energy, one obtains the potential energy for the k-th electronic eigenstate of the system

(3) $$\begin{array}{l} U_{k}=E_{k}+\frac{e^{2}}{4\pi \epsilon_{0}}\overset{N}{\underset{A=1}{\sum }}\overset{N}{\underset{B>A}{\sum }}\frac{Z_{A}Z_{B}}{\left|{\text{R}}_{A}-{\text{R}}_{B}\right|}. \end{array}$$

By varying the nuclear positions and solving the corresponding time-independent electronic Schrödinger equation, *U*_*k*_ can be expressed as a function of the nuclear positions, also known as the potential energy surface of the k-th electronic eigenstate. In the second step of the BO approximation, the kinetic energy of the nuclei is reintroduced, and in principle, the nuclear dynamics is described by the time-dependent nuclear Schrödinger equation, evolving on a potential energy surface, e.g., *U*_*k*_(**R**_1_, …, **R**_*N*_).

Similar to most AIMD simulations (Tuckerman, 2002; Jensen, 2007; Marx and Hutter, 2009; Gaigeot, 2010; Ramírez-Solís et al., 2011; Vitale et al., 2015), in this work, we assume that nuclear quantum effects could be ignored, and nuclear motion occurs only on the GS potential energy surface (i.e., the potential energy surface of the electronic GS), *U*_0_(**R**_1_, …, **R**_*N*_). Since the time-independent electronic Schrödinger equation is only applicable to very small electronic systems, a sufficiently efficient electronic structure method for the determination of electronic ground state is typically required for AIMD simulations. As mentioned previously, KS-AIMD simulations can be unreliable for electronic systems with a radical nature. On the other hand, AIMD simulations employing MR electronic structure methods are very likely to be computationally intractable for most electronic systems.

To resolve this issue with minimum computational expense, in the present work, we combine TAO-DFT (Chai, 2012) with AIMD, yielding the TAO-AIMD method. Specifically, in TAO-AIMD, the nuclei are treated as classical particles, obeying the classical nuclear Hamiltonian

(4) $$\begin{array}{l} H_{\text{nucl}}\left({\text{P}}_{1}\left(t\right),...,{\text{P}}_{N}\left(t\right),{\text{R}}_{1}\left(t\right),...,{\text{R}}_{N}\left(t\right)\right)=\overset{N}{\underset{A=1}{\sum }}\frac{|{\text{P}}_{A}\left(t\right){|}^{2}}{2M_{A}}\\ \;+U_{0}^{\text{TAO-DFT}}\left({\text{R}}_{1}\left(t\right),...,{\text{R}}_{N}\left(t\right)\right), \end{array}$$

where *M*_*A*_ is the mass of nucleus *A*, and **P**_*A*_(*t*) is the momentum of nucleus *A* at time *t*. The left-hand side of Equation (4) gives the total energy at time *t* [i.e., *E*(*t*)]. On the right-hand side of Equation (4), the first term is the nuclear kinetic energy at time *t*, and the second term is the potential energy of the electronic GS obtained with TAO-DFT for the nuclear positions at time *t*, i.e., **R**_1_(*t*), …, **R**_*N*_(*t*):

(5) $$\begin{array}{l} U_{0}^{\text{TAO-DFT}}\left({\text{R}}_{1}\left(t\right),...,{\text{R}}_{N}\left(t\right)\right)=E_{0}^{\text{TAO-DFT}}\left({\text{R}}_{1}\left(t\right),...,{\text{R}}_{N}\left(t\right)\right)\\ \;+\frac{e^{2}}{4\pi \epsilon_{0}}\overset{N}{\underset{A=1}{\sum }}\overset{N}{\underset{B>A}{\sum }}\frac{Z_{A}Z_{B}}{\left|{\text{R}}_{A}\left(t\right)-{\text{R}}_{B}\left(t\right)\right|}, \end{array}$$

where $E_{0}^{\text{TAO-DFT}}\left({\text{R}}_{1}\left(t\right),...,{\text{R}}_{N}\left(t\right)\right)$ (e.g., see Equation 27 of Chai, 2012) is the corresponding GS electronic energy obtained with TAO-DFT. On the basis of Equation (4), the nuclei move based on Newton's equations of motion on the GS potential energy surface generated by TAO-DFT:

(6) $$\begin{array}{l} {\dot{\text{R}}}_{A}\left(t\right)=\frac{{\text{P}}_{A}\left(t\right)}{M_{A}} \end{array}$$

(7) $$\begin{array}{l} {\dot{\text{P}}}_{A}\left(t\right)=-\nabla_{A}U_{0}^{\text{TAO-DFT}}\left({\text{R}}_{1}\left(t\right),...,{\text{R}}_{N}\left(t\right)\right), \end{array}$$

where ${\dot{\text{R}}}_{A}\left(t\right)$ is the velocity of nucleus *A* at time *t*, ${\dot{\text{P}}}_{A}\left(t\right)$ is the time derivative of the momentum of nucleus *A* at time *t*, and the right-hand side of Equation (7) gives the force acting on nucleus *A* at time *t* [i.e., **F**_*A*_(*t*)].

Equations (4)–(7) form the theoretical basis of the TAO-AIMD method. Given the initial nuclear positions **R**_1_(0), …, **R**_*N*_(0) and velocities ${\dot{\text{R}}}_{1}\left(0\right),...,{\dot{\text{R}}}_{N}\left(0\right)$, all the future nuclear positions **R**_1_(*t*), …, **R**_*N*_(*t*) and velocities ${\dot{\text{R}}}_{1}\left(t\right),...,{\dot{\text{R}}}_{N}\left(t\right)$ are determined by Equations (5)–(7), generating a TAO-AIMD trajectory (i.e., TAO-AIMD is deterministic). Note that the GS potential energy surface and the forces that act on the nuclei can be computed on-the-fly using TAO-DFT, as needed along the TAO-AIMD trajectory. According to the definitions presented here (Marx and Hutter, 2009), TAO-AIMD can also be regarded as TAO-DFT-based Born-Oppenheimer MD (BOMD), i.e., TAO-BOMD.

Note that the GS potential energy surface and the forces that act on the nuclei are computed using TAO-DFT in TAO-AIMD, while they are computed using KS-DFT in KS-AIMD. Since TAO-DFT is as computationally efficient as KS-DFT (e.g., see Chai, 2012 for details), TAO-AIMD is similar to KS-AIMD in computational expense. On the other hand, AIMD simulations employing MR electronic structure methods are very likely to be computationally infeasible for most electronic systems. Accordingly, TAO-AIMD can be a very promising method for exploring the equilibrium thermodynamic and dynamical properties of nanosystems with a radical nature at finite temperatures. In addition, existing XC energy functionals defined in KS-DFT could also be employed in TAO-DFT (Chai, 2012, 2014, 2017) and TAO-AIMD. For electronic systems with a nonradical nature, TAO-DFT performs similarly to KS-DFT, and TAO-AIMD should therefore perform similarly to KS-AIMD.

## 3. Computational Details

All calculations are performed using TAO-LDA (Chai, 2012), i.e., TAO-DFT employing the LDA XC and θ-dependent energy functionals, where the suggested fictitious temperature θ = 7 mhartree is employed (Chai, 2012). Almost all numerical data are obtained with Q-Chem 4.4 (Shao, 2015), using the 6-31G(d) basis set and a numerical grid consisting of 75 Euler-Maclaurin radial grid points and 302 Lebedev angular grid points, wherein isolated boundary conditions (i.e., well-suited for the study of atoms and molecules) are employed. The TAO-AIMD-based IR spectra are computed using the program TRAVIS (Brehm and Kirchner, 2011; Thomas et al., 2013, 2015; Brehm et al., 2020).

In several recent investigations (Chai, 2012; Wu and Chai, 2015; Fosso-Tande et al., 2016; Yeh and Chai, 2016; Mullinax et al., 2019), the orbital occupation numbers obtained from TAO-LDA (with θ = 7 mhartree) have been found to be similar to the natural orbital occupation numbers obtained from a very accurate MR electronic structure method that can be applied to treat relatively large active spaces, leading to a qualitatively similar tendency for the radical nature associated with various PAHs (i.e., polycyclic aromatic hydrocarbons).

On the other hand, because of the constraint of symmetry, the spin-unrestricted and spin-restricted calculations based on an exact electronic structure method should lead to the same energy values for the lowest singlet state (i.e., GS) of *n*-acene (Chai, 2012; Rivero et al., 2013; Gryn'ova et al., 2015). Nevertheless, KS-DFT employing conventional XC energy functionals fails to obey this constraint for the larger *n*-acenes (which are electronic systems possessing radical nature), leading to the unphysical symmetry-breaking effects in the corresponding spin-unrestricted calculations (Cohen et al., 2008, 2012). In our previous studies (Chai, 2012, 2014; Wu and Chai, 2015), the spin-unrestricted and spin-restricted GS (i.e., lowest singlet state) energy values of *n*-acene (up to *n* = 100), calculated by TAO-LDA (with θ = 7 mhartree), have been found to be essentially the same, leading to essentially no unphysical symmetry-breaking effects in the corresponding spin-unrestricted calculations. For computational efficiency, therefore, all TAO-LDA calculations in this work are spin-restricted calculations unless noted otherwise.

For all TAO-AIMD simulations, a time step of 20 a.u. (≈ 0.484 fs) is adopted for the integration of the equations of motion. For each TAO-AIMD simulation, the initial geometry of *n*-acene is chosen as the GS geometry of *n*-acene obtained with TAO-LDA, and the initial nuclear velocities of *n*-acene are randomly selected from the Maxwell-Boltzmann (MB) distribution at *T* = 300 K. To equilibrate *n*-acene at *T* = 300 K, the TAO-AIMD simulation is first performed in the canonical (*NVT*) ensemble with the aid of the Nosé-Hoover (NH) chain thermostat (Martyna et al., 1992) (with the NH chain length of three auxiliary variables and NH timescale of 200 fs in the implementation of Q-Chem) for about 10.2 ps. To avoid any interference with the dynamics (Gaigeot and Sprik, 2003), we subsequently remove the NH chain thermostat and continue the TAO-AIMD simulation in the microcanonical (*NVE*) ensemble for a total of 10,500 time steps (≈ 5.1 ps), at which time the total energy of *n*-acene is well converged. After equilibration, we continue the TAO-AIMD simulation in the *NVE* ensemble and collect relevant data along the equilibrated TAO-AIMD trajectory for a total of 42,000 time steps (≈ 20.3 ps), where the average temperature is 300 ± 1 K. As the initial nuclear velocities of *n*-acene for each TAO-AIMD simulation are *randomly selected* from the MB distribution at 300 K, the aforementioned processes are repeated to generate a total of four different equilibrated TAO-AIMD trajectories (≈ 20.3 ps per trajectory).

Furthermore, to demonstrate the significance of TAO-AIMD simulations for exploring the dynamical information of large molecules with radical nature, we also perform preliminary calculations on 8-acene to examine the possible symmetry-breaking effects in the spin-unrestricted TAO-AIMD/KS-AIMD simulations (see Supplementary Section 1 and Supplementary Figures 1–3).

## 4. Results and Discussion

### 4.1. Symmetrized von Neumann Entropy

In TAO-DFT, the radical nature of a GS molecule can be examined by the symmetrized von Neumann entropy (for example, see Equation 5 of Chung and Chai, 2019 for the spin-unrestricted case). For the spin-restricted case, the symmetrized von Neumann entropy of a GS molecule can be expressed as

(8) $$\begin{array}{l} S_{\text{vN}}=-\overset{\infty }{\underset{i=1}{\sum }}\left\{\frac{f_{i}}{2}\;\text{ln}\left(\frac{f_{i}}{2}\right)+\left(1-\frac{f_{i}}{2}\right)\;\text{ln}\left(1-\frac{f_{i}}{2}\right)\right\}, \end{array}$$

where *f*_*i*_ (i.e., a number between 0 and 2) is the occupation number of the i-th orbital of the GS molecule, obtained with spin-restricted TAO-DFT. Note that *f*_*i*_ is closely related to the corresponding natural orbital occupation number (Löwdin and Shull, 1956; Chai, 2012, 2014, 2017). For a GS molecule possessing a nonradical nature, the occupation numbers associated with all orbitals are very close to either 0 or 2, yielding a vanishingly small *S*_vN_ value. Nonetheless, for a GS molecule with a significant radical nature, the active orbital occupation numbers can deviate significantly from 0 and 2 (for example, 0.2–1.8); hence, the corresponding *S*_vN_ value can greatly increase as the number of active orbitals increases and/or the active orbital occupation numbers are closer to 1 (Rivero et al., 2013; Chai, 2014, 2017; Wu and Chai, 2015; Seenithurai and Chai, 2016, 2017, 2018, 2019; Wu et al., 2016; Yeh et al., 2018; Chung and Chai, 2019; Deng and Chai, 2019; Huang et al., 2020).

On the basis of Equation (8), in a spin-restricted TAO-AIMD simulation, the symmetrized von Neumann entropy of a molecule at time *t* along a TAO-AIMD trajectory can be defined as

(9) $$\begin{array}{l} S_{\text{vN}}\left(t\right)=-\overset{\infty }{\underset{i=1}{\sum }}\left\{\frac{f_{i}\left(t\right)}{2}\;\text{ln}\left(\frac{f_{i}\left(t\right)}{2}\right)+\left(1-\frac{f_{i}\left(t\right)}{2}\right)\;\text{ln}\left(1-\frac{f_{i}\left(t\right)}{2}\right)\right\}, \end{array}$$

where *f*_*i*_(*t*) (i.e., a value between 0 and 2) is the occupation number of the *i*^th^ orbital of the molecule, obtained with spin-restricted TAO-DFT for the nuclear positions at time *t*, i.e., **R**_1_(*t*), …, **R**_*N*_(*t*), along the TAO-AIMD trajectory. Accordingly, the time average of *S*_vN_(*t*) along the TAO-AIMD trajectory is calculated by

(10) $$\begin{array}{l} \bar{S_{\text{vN}}}=\frac{1}{\tau }\int_{0}^{\tau }S_{\text{vN}}\left(t\right)dt, \end{array}$$

where τ is the total time duration of the TAO-AIMD trajectory.

To investigate the radical nature of *n*-acene in the TAO-AIMD simulations, the $\bar{S_{\text{vN}}}$ value of *n*-acene along each equilibrated TAO-AIMD trajectory is computed using Equation (10), and the reported $\bar{S_{\text{vN}}}$ value of *n*-acene is an average over four different equilibrated TAO-AIMD trajectories (≈ 20.3 ps per trajectory). For comparison, we also report the *S*_vN_ value of GS *n*-acene (given by Equation 8), corresponding to the $\bar{S_{\text{vN}}}$ value of *n*-acene at 0 K in this work.

**Figure 2:**
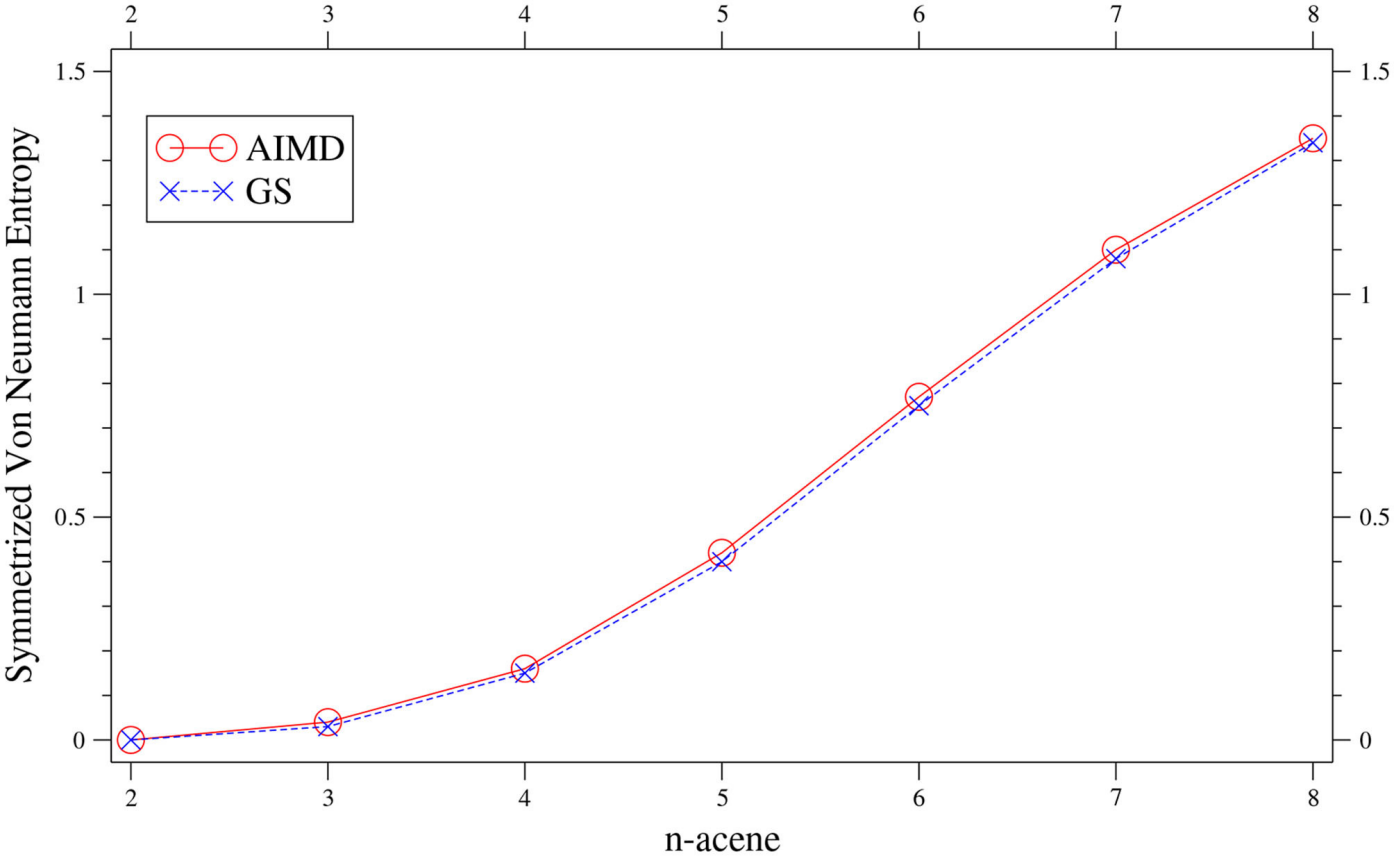
Symmetrized von Neumann entropy of *n*-acene, obtained with the TAO-AIMD simulations at 300 K and GS calculation, calculated by TAO-LDA.

As presented in Figure 2, the $\bar{S_{\text{vN}}}$ value of *n*-acene obtained with the TAO-AIMD simulations at 300 K is very close to the *S*_vN_ value of GS *n*-acene Chai (2014), increasing monotonically with increasing *n* (also see Supplementary Table 1). This suggests that similar to the GS counterparts at 0 K (Hachmann et al., 2007; Chai, 2012, 2014, 2017), on average, the smaller *n*-acenes (up to *n* = 5) possess nonradical nature, and the larger *n*-acenes (*n* = 6–8) possess increasing radical nature in AIMD simulations at 300 K.

**Figure 3:**
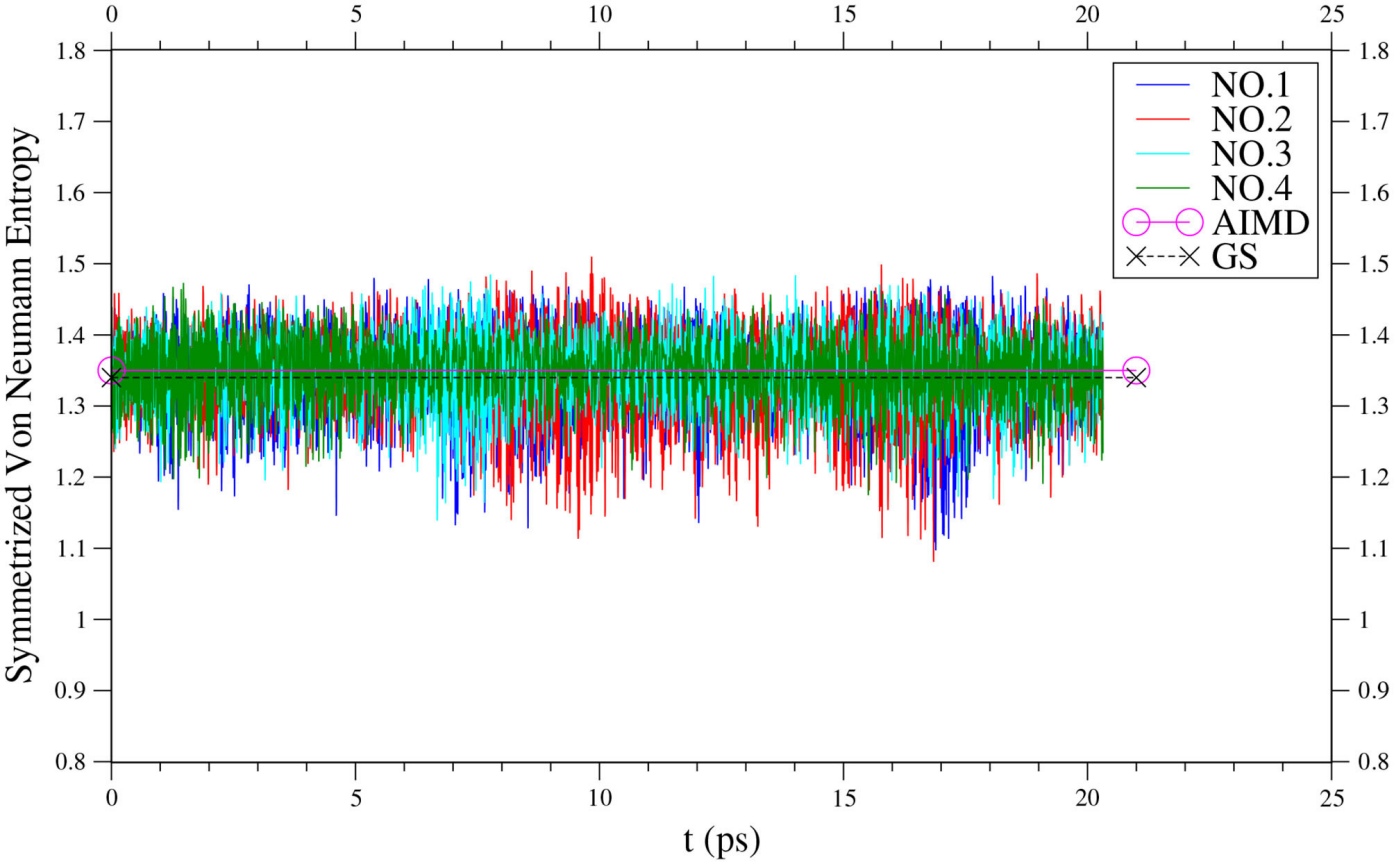
Time evolution of the symmetrized von Neumann entropy of 8-acene, obtained from four different equilibrated TAO-AIMD trajectories (No.1 to No.4) at 300 K, calculated by TAO-LDA. The TAO-AIMD average and GS values are also shown for comparison.

Along each equilibrated TAO-AIMD trajectory, the instantaneous *S*_vN_(*t*) value of *n*-acene fluctuates over time and can thus be even larger than the $\bar{S_{\text{vN}}}$ value of *n*-acene (see Figure 3 for *n* = 8, and Supplementary Figures 4–9 for others). Therefore, for the larger *n*-acenes (*n* = 6–8), it is essential to perform AIMD simulations at 300 K with an efficient electronic structure method that can reliably describe strong static correlation effects, well-justifying the use of TAO-AIMD in this work.

### 4.2. Active Orbital Occupation Numbers

As mentioned previously, in a spin-restricted TAO-AIMD simulation, *f*_*i*_(*t*) (i.e., a value between 0 and 2) is the occupation number of the i-th orbital of a molecule, obtained with spin-restricted TAO-DFT for the nuclear positions at time *t*, i.e., **R**_1_(*t*), …, **R**_*N*_(*t*), along the TAO-AIMD trajectory. Consequently, the time average of *f*_*i*_(*t*) along the TAO-AIMD trajectory is calculated by

(11) $$\begin{array}{l} \bar{f_{i}}=\frac{1}{\tau }\int_{0}^{\tau }f_{i}\left(t\right)dt, \end{array}$$

where τ is the total time duration of the TAO-AIMD trajectory.

To further assess the radical nature of *n*-acene in the TAO-AIMD simulations, the $\bar{f_{i}}$ value of *n*-acene along each equilibrated TAO-AIMD trajectory is computed using Equation (11), and the reported $\bar{f_{i}}$ value of *n*-acene is an average over four different equilibrated TAO-AIMD trajectories (≈ 20.3 ps per trajectory). For comparison, we also present the *f*_*i*_ value of GS *n*-acene, which corresponds to the $\bar{f_{i}}$ value of *n*-acene at 0 K in this work.

For *n*-acene (containing *N*_*e*_ electrons), we define the HOMO (i.e., highest occupied molecular orbital) as the (*N*_*e*_/2)th orbital, the LUMO (i.e., lowest unoccupied molecular orbital) as the (*N*_*e*_/2 + 1)th orbital, and so forth (Chai, 2012, 2017; Wu and Chai, 2015; Wu et al., 2016; Yeh et al., 2018; Chung and Chai, 2019; Deng and Chai, 2019; Seenithurai and Chai, 2019; Huang et al., 2020). For brevity, HOMO and LUMO are denoted as H and L, respectively. In addition, the orbitals with an occupation number (0.2–1.8) are regarded as the active orbitals.

**Figure 4:**
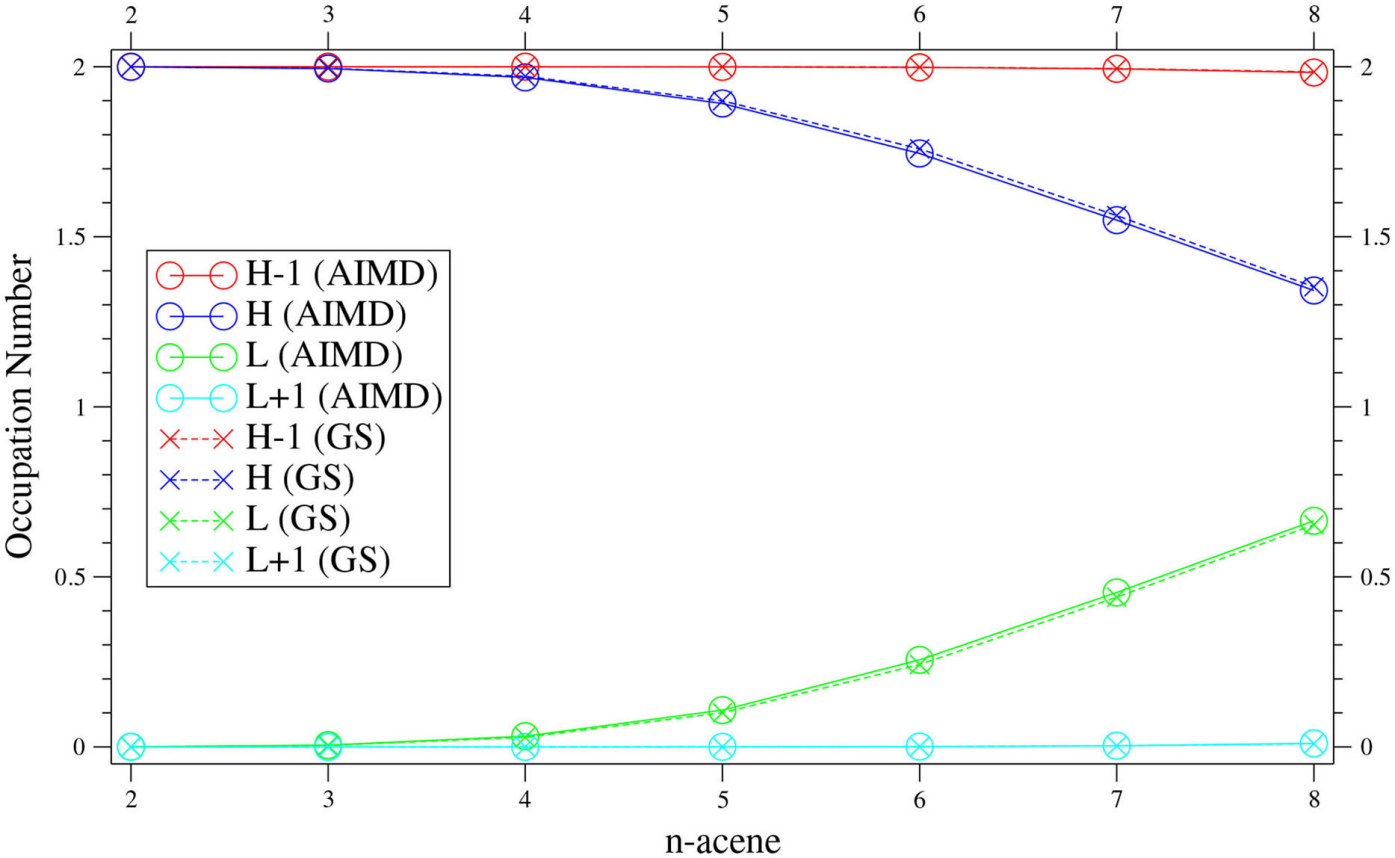
Active orbital occupation numbers (HOMO−1, HOMO, LUMO, and LUMO+1) of *n*-acene, obtained with the TAO-AIMD simulations at 300 K and GS calculation, calculated by TAO-LDA.

As presented in Figure 4, the $\bar{f_{i}}$ value of *n*-acene obtained with the TAO-AIMD simulations at 300 K is very close to the *f*_*i*_ value of GS *n*-acene (Chai, 2012) (also see Supplementary Table 2). This implies that on average, the radical nature of *n*-acene obtained with the TAO-AIMD simulations at 300 K is very similar to that obtained with the GS calculation at 0 K, showing consistency with the analysis of symmetrized von Neumann entropy. For smaller *n* values (e.g., up to *n* = 5), the occupation numbers associated with all orbitals are very close to either 0 or 2. Therefore, the smaller *n*-acenes should exhibit nonradical nature. However, as *n* increases, the number of active orbitals increases and/or the active orbital occupation numbers are closer to 1, apparently showing that the larger *n*-acenes (*n* = 6–8) should exhibit increasing radical character. This clearly indicates that similar to the GS counterparts at 0 K (Hachmann et al., 2007; Chai, 2012, 2014, 2017), on average, the larger *n*-acenes (*n* = 6–8) should possess increasing radical nature in AIMD simulations at 300 K.

**Figure 5:**
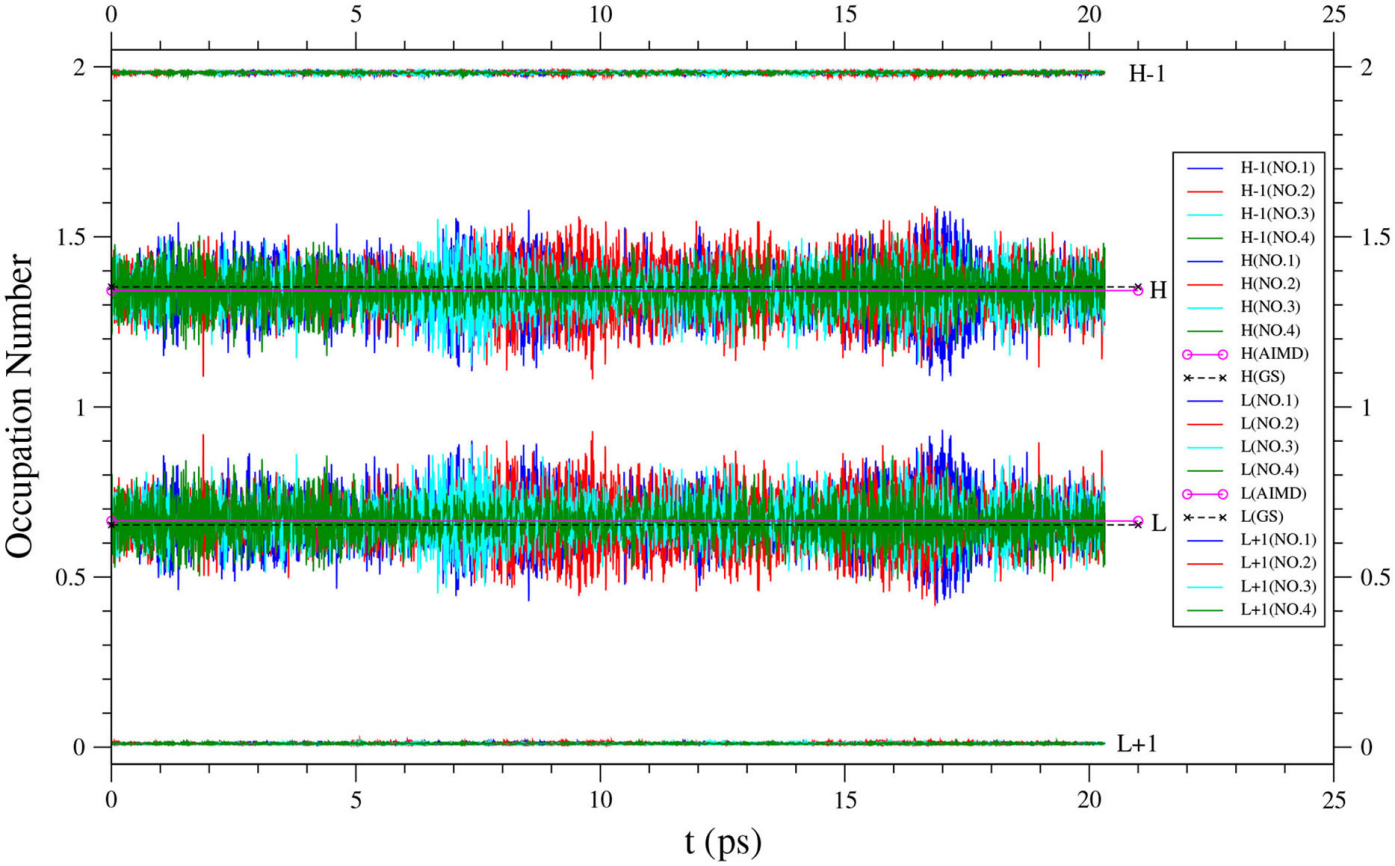
Time evolution of the active orbital occupation numbers (HOMO−1, HOMO, LUMO, and LUMO+1) of 8-acene, obtained from four different equilibrated TAO-AIMD trajectories (No.1 to No.4) at 300 K, calculated by TAO-LDA. For HOMO/LUMO, the TAO-AIMD average and GS values are also shown for comparison.

In addition, the instantaneous *f*_*i*_(*t*) value of *n*-acene along each equilibrated TAO-AIMD trajectory fluctuates around an average, implying that the instantaneous radical nature of *n*-acene can be more pronounced than the average radical nature of *n*-acene in the TAO-AIMD simulations (see Figure 5 for *n* = 8, and Supplementary Figures 10–15 for others). According to our findings, the radical nature of the larger *n*-acenes (*n* = 6–8) can persist in AIMD simulations at 300 K. For such molecules, KS-AIMD simulations can therefore be unreliable, and AIMD simulations employing MR electronic structure methods are very likely to be computationally intractable. Accordingly, this highlights the significance of TAO-AIMD simulations for exploring the dynamical information of large molecules with radical nature.

### 4.3. IR Spectra

IR spectroscopy, which involves the interaction of IR radiation with matter, has been extensively used to explore the structure of materials nondestructively in recent years (Roggo et al., 2007; Joblin et al., 2011; Beć and Huck, 2019). Recently, the IR spectra of PAHs (e.g., *n*-acenes) have attracted much interest since PAHs can be responsible for the unidentified infrared emission (UIR) bands from interstellar media (Allamandola et al., 1985, 1999; Hudgins and Sandford, 1998a,b; Kim et al., 2001; Joblin et al., 2011).

On the basis of the Fermi golden rule of time-dependent perturbation theory (McQuarrie, 1976), the IR spectrum of a molecule is related to the Fourier transform of the autocorrelation function (ACF) (e.g., see Equation 15 of Brehm et al., 2020) of the dipole moment (i.e., the sum of the electronic and nuclear contributions) $\vec{\mu }$ of the molecule (Thomas et al., 2013, 2015; Dutta and Chowdhury, 2019):

(12) $$\begin{array}{l} I\left(\omega \right)\propto \omega^{2}\int_{-\infty }^{\infty }\langle \vec{\mu }\left(0\right)\cdot \vec{\mu }\left(t\right)\rangle e^{-i\omega t}dt, \end{array}$$

where *I*(ω) is the IR intensity, and ω is the vibrational frequency. Note that a quantum correction factor *βℏω*/(1−*e*^−*βℏω*^) (Ramírez et al., 2004; Joalland et al., 2010; Thomas et al., 2013) has been taken into account in Equation (12), where β = 1/(*k*_*B*_*T*) and *k*_*B*_ is the Boltzmann constant. According to the properties of the Fourier transform (Thomas et al., 2013; Lawson Daku, 2018), *I*(ω) can also be expressed as

(13) $$\begin{array}{l} I\left(\omega \right)\propto \int_{-\infty }^{\infty }\langle \dot{\vec{\mu }}\left(0\right)\cdot \dot{\vec{\mu }}\left(t\right)\rangle e^{-i\omega t}dt, \end{array}$$

which is directly proportional to the Fourier transform of the ACF of the time derivative of the dipole moment $\dot{\vec{\mu }}$ of the molecule.

Given the $\vec{\mu }\left(t\right)$ of *n*-acene along an equilibrated TAO-AIMD trajectory (obtained with Q-Chem 4.4, Shao, 2015), we compute the IR spectrum of *n*-acene using the TRAVIS program package (Brehm and Kirchner, 2011; Thomas et al., 2013, 2015; Brehm et al., 2020). To reduce the numerical noise, the IR spectrum has been smoothed using a window function applied in the time domain [specifically, each term of the ACF is multiplied by a Gaussian function $\text{exp}\left(-\frac{\sigma t^{2}}{2\tau^{2}}\right)$, where σ = 10 (as suggested in Gaigeot et al., 2005; Gaigeot, 2010; Vitale et al., 2015 for gas phase simulations) and τ is the total time duration of the equilibrated TAO-AIMD trajectory (≈ 20.3 ps)]. The reported IR spectrum of *n*-acene is an average over four different equilibrated TAO-AIMD trajectories (with the average temperature being 300 ± 1 K).

On the other hand, NMA (i.e., normal mode analysis) is a commonly adopted approach to compute the vibrational frequencies and intensities of GS (Harris and Bertolucci, 1978; Wilson et al., 1980; Gaigeot et al., 2007) and excited-state (ES) (Liu and Liang, 2011a,b) molecules in the harmonic approximation at 0 K. For comparison purposes, we also compute the IR spectra of GS *n*-acenes using NMA. To perform a GS-NMA, the computation of nuclear second derivatives of energy (i.e., the nuclear Hessian) at the GS molecular geometry is required. Since analytical nuclear Hessians for TAO-DFT are not yet available in Q-Chem, numerical nuclear Hessians are computed using finite differences of analytical nuclear gradients (with a step size of 0.001 Å, i.e., the default setting of Q-Chem) for all the GS-NMA performed in this work.

**Figure 6:**
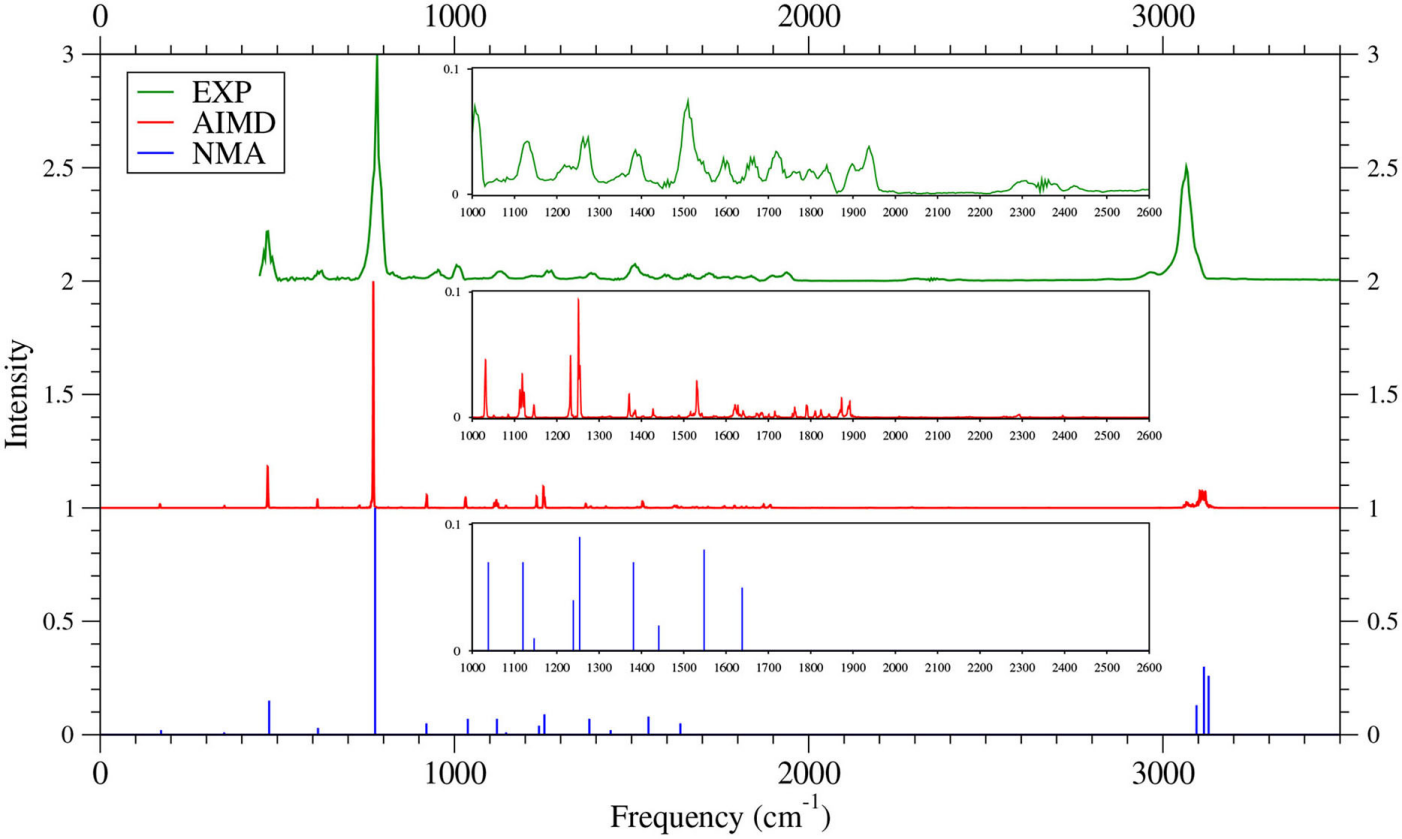
IR spectra of 2-acene, obtained with the TAO-AIMD simulations at 300 K and GS-NMA, calculated by TAO-LDA. Experimental (EXP) data (NIST mass spectrometry data center, 2020) are included for comparison. The IR spectra are normalized to have a maximum intensity of one and, for clarity, are vertically offset from each other by the same value. Subfigures show the IR spectra in the 1,000–2,600 cm^−1^ range.

**Figure 7:**
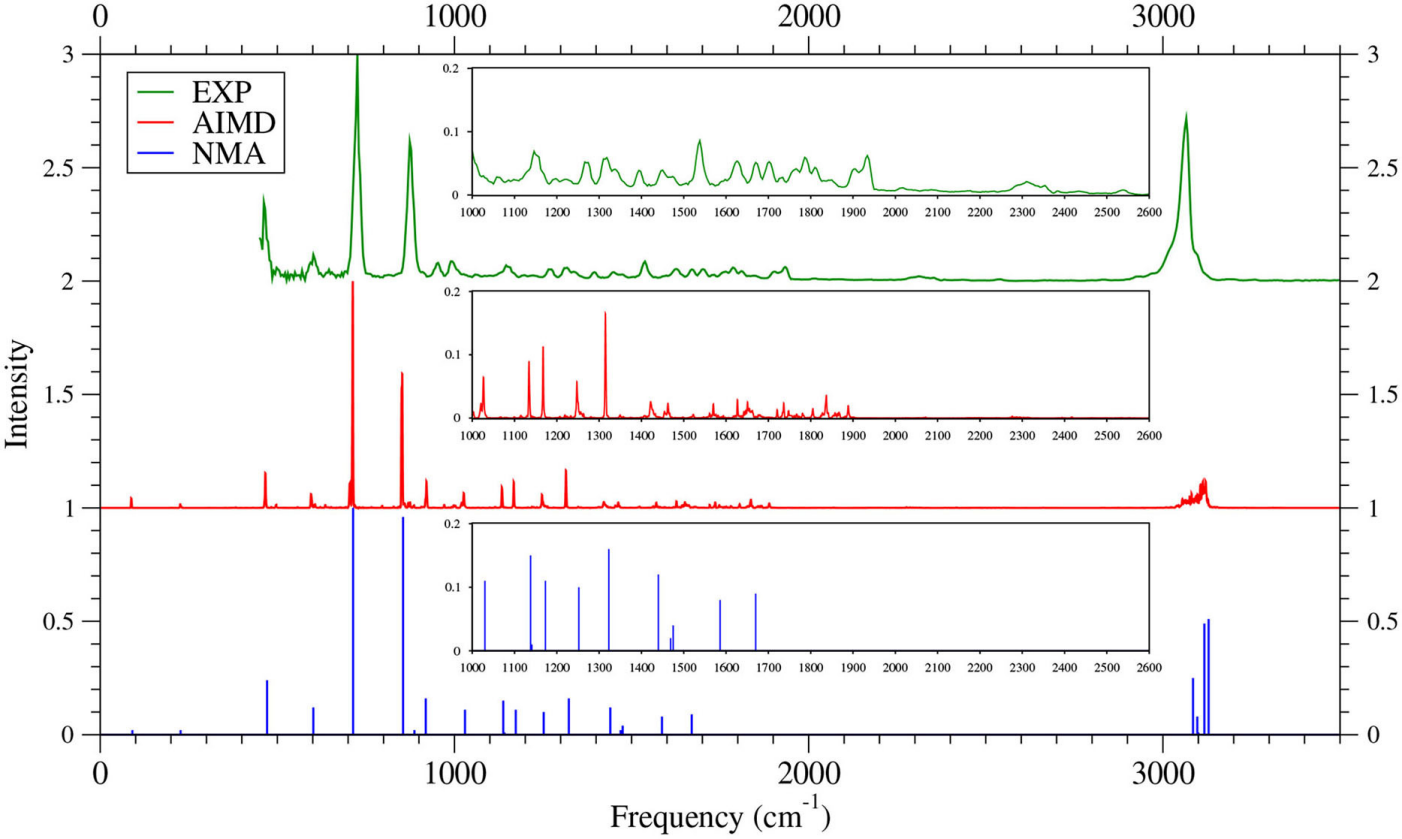
IR spectra of 3-acene, obtained with the TAO-AIMD simulations at 300 K and GS-NMA, calculated by TAO-LDA. Experimental (EXP) data NIST mass spectrometry data center (2020) are included for comparison. The IR spectra are normalized to have a maximum intensity of one and, for clarity, are vertically offset from each other by the same value. Subfigures show the IR spectra in the 1,000–2,600 cm^−1^ range.

**Figure 8:**
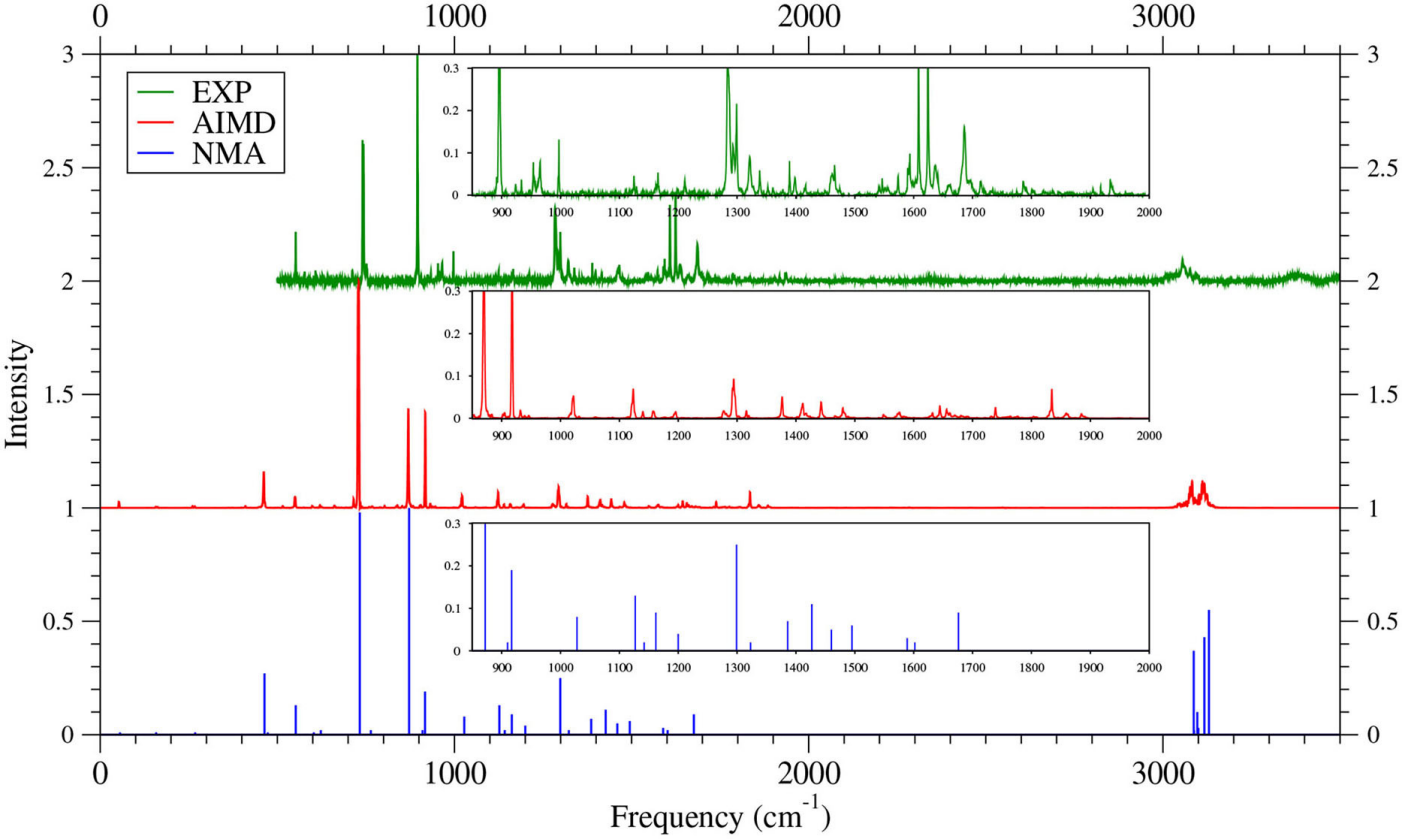
IR spectra of 4-acene, obtained with the TAO-AIMD simulations at 300 K and GS-NMA, calculated by TAO-LDA. Experimental (EXP) data (Hudgins and Sandford, 1998a; Boersma et al., 2014; Bauschlicher et al., 2018; Mattioda et al., unpublished) are included for comparison. The IR spectra are normalized to have a maximum intensity of one and, for clarity, are vertically offset from each other by the same value. Subfigures show the IR spectra in the 850–2,000 cm^−1^ range.

**Figure 9:**
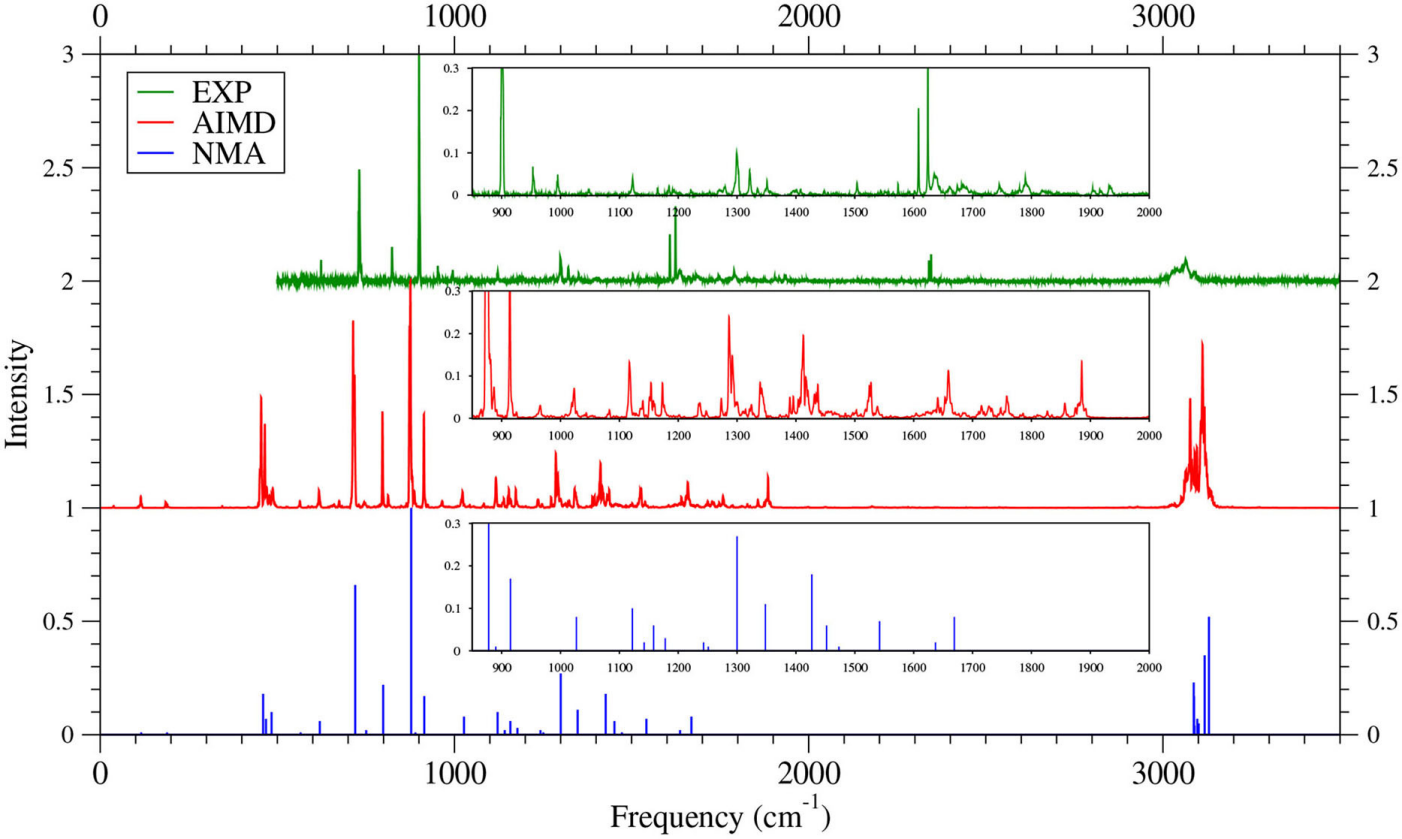
IR spectra of 5-acene, obtained with the TAO-AIMD simulations at 300 K and GS-NMA, calculated by TAO-LDA. Experimental (EXP) data (Hudgins and Sandford, 1998b; Boersma et al., 2014; Bauschlicher et al., 2018; Mattioda et al., unpublished) are included for comparison. The IR spectra are normalized to have a maximum intensity of one and, for clarity, are vertically offset from each other by the same value. Subfigures show the IR spectra in the 850–2,000 cm^−1^ range.

As shown in Figures 6–9 (for comparison purposes, also see Supplementary Figures 16–19 for the TAO-AIMD results obtained with different values of σ), the IR spectra of the smaller *n*-acenes (*n* = 2–5), obtained with the TAO-AIMD simulations at 300 K and GS-NMA, are in qualitative agreement with the available experimental IR spectra (Hudgins and Sandford, 1998a,b; Boersma et al., 2014; Bauschlicher et al., 2018; NIST mass spectrometry data center, 2020; Mattioda et al., unpublished). Note that a number of experimental IR bands (e.g., those in the 1,700–2,600 cm^−1^ range for 2-acene and 3-acene and those in the 1,700–2,000 cm^−1^ range for 4-acene and 5-acene) are completely missing from the corresponding IR spectra obtained with the commonly used GS-NMA approach, indicating that anharmonic effects on the IR spectra cannot be completely ignored. By contrast, these IR bands can be obtained with the TAO-AIMD simulations at 300 K, going beyond the harmonic approximation of GS-NMA. The overall discrepancies between theoretical and experimental results for the IR spectra can be attributed to several factors, such as the purity of samples and environmental factors (temperature, background noise level, etc.) in the experiments and the approximate nature of the theoretical methods adopted.

**Figure 10:**
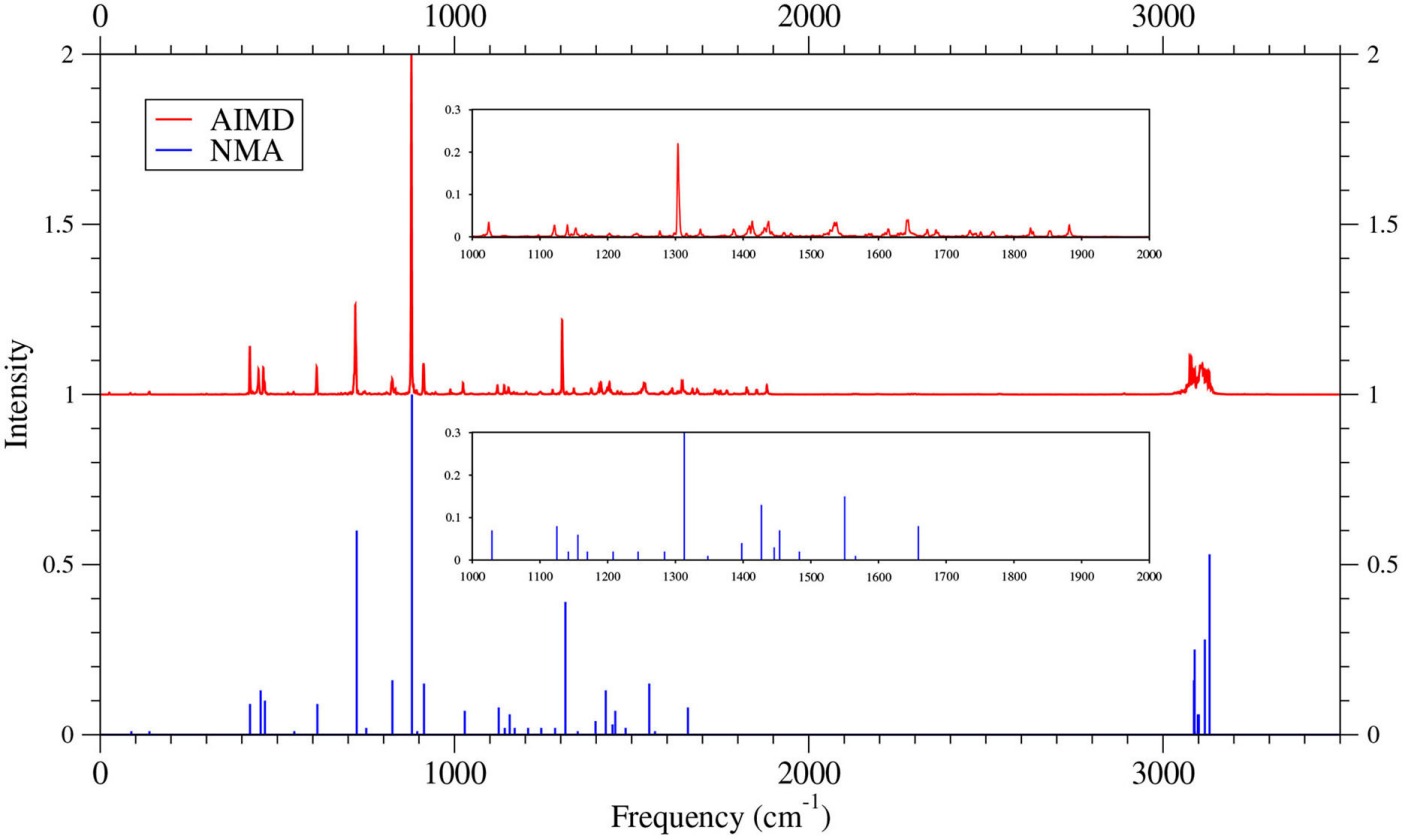
IR spectra of 6-acene, obtained with the TAO-AIMD simulations at 300 K and GS-NMA, calculated by TAO-LDA. The IR spectra are normalized to have a maximum intensity of one and, for clarity, are vertically offset from each other by the same value. Subfigures show the IR spectra in the 1,000–2,000 cm^−1^ range.

**Figure 11:**
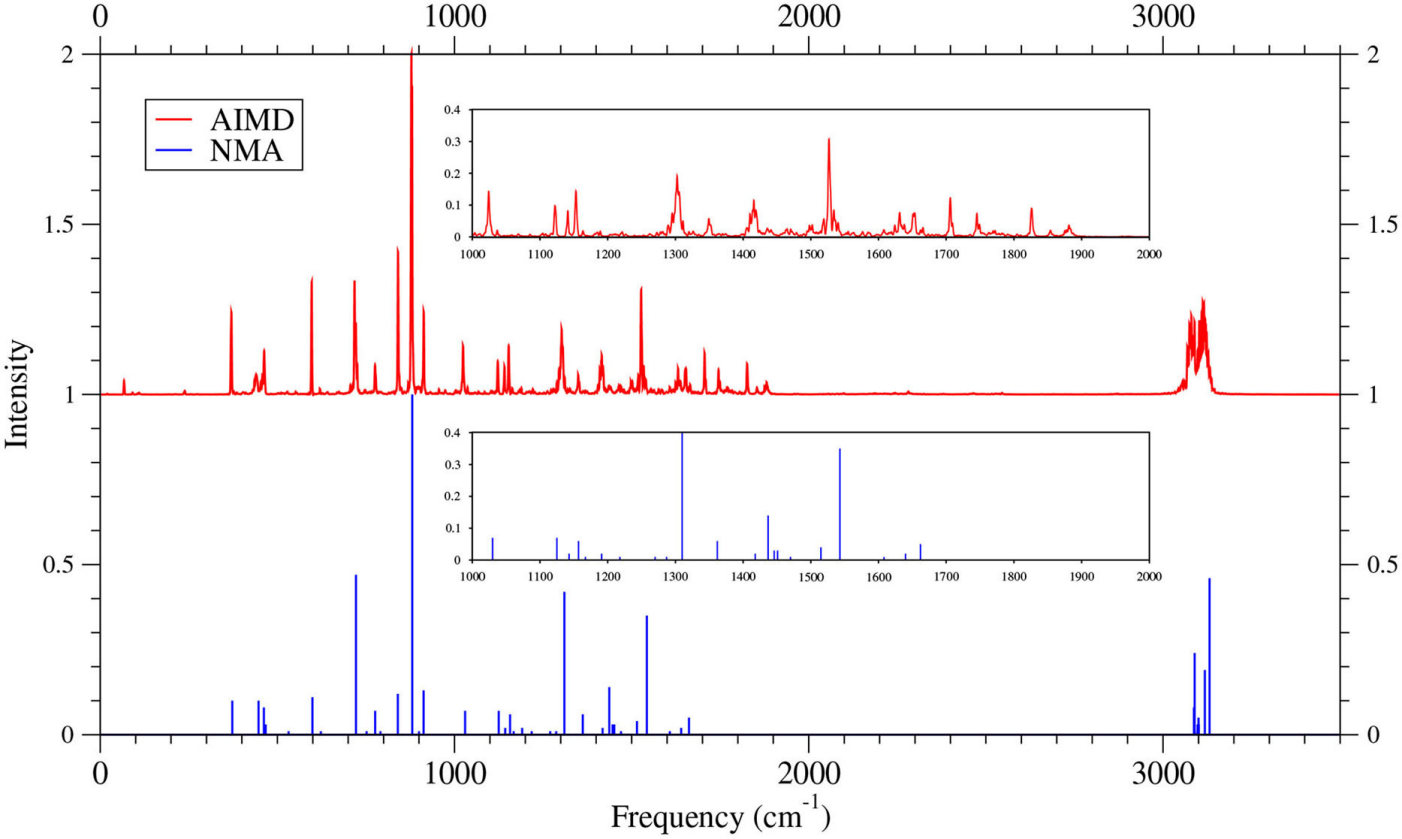
IR spectra of 7-acene, obtained with the TAO-AIMD simulations at 300 K and GS-NMA, calculated by TAO-LDA. The IR spectra are normalized to have a maximum intensity of one and, for clarity, are vertically offset from each other by the same value. Subfigures show the IR spectra in the 1,000–2,000 cm^−1^ range.

**Figure 12:**
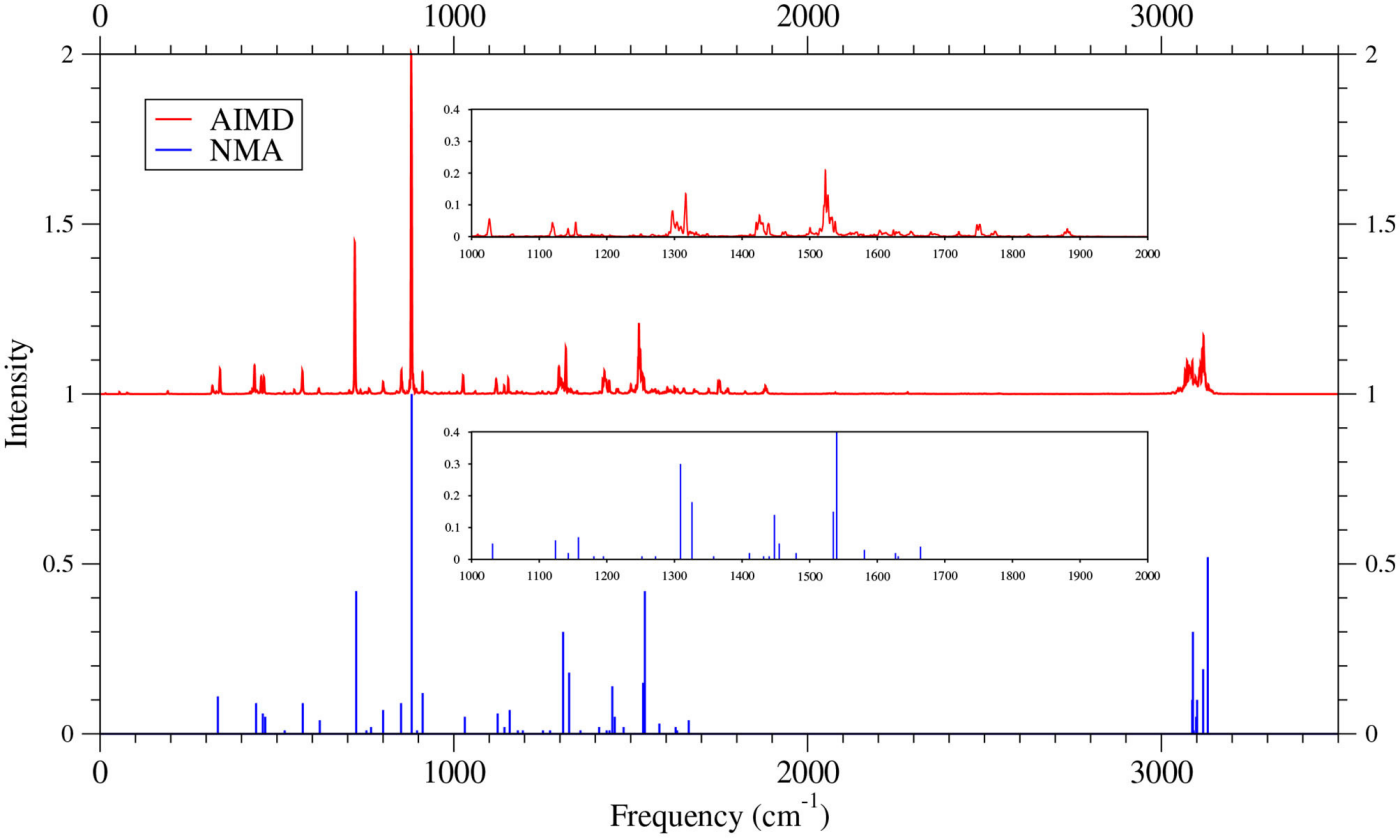
IR spectra of 8-acene, obtained with the TAO-AIMD simulations at 300 K and GS-NMA, calculated by TAO-LDA. The IR spectra are normalized to have a maximum intensity of one and, for clarity, are vertically offset from each other by the same value. Subfigures show the IR spectra in the 1,000–2,000 cm^−1^ range.

To our knowledge, there are no experimental IR spectra for 6-acene, 7-acene, and 8-acene. It remains highly challenging to synthesize and isolate the larger *n*-acenes (*n* = 6–8), possibly due to their radical character (Hachmann et al., 2007; Chai, 2012, 2014, 2017; Rivero et al., 2013; Wu and Chai, 2015; Fosso-Tande et al., 2016). In Figures 10–12 (for comparison purposes, also see Supplementary Figures 20–22 for the TAO-AIMD results obtained with different values of σ), we thus only compare the IR spectra of the larger *n*-acenes (*n* = 6–8), obtained with the TAO-AIMD simulations at 300 K and GS-NMA. As shown, both theoretical results are in reasonably good agreement, except for the 1,700–2,000 cm^−1^ range, which may be attributed to anharmonic effects on the IR spectra [cf., the IR spectra of the smaller *n*-acenes (*n* = 2–5)].

## 5. Conclusions

In conclusion, we have proposed TAO-AIMD (i.e., TAO-DFT-based AIMD) for the study of the equilibrium thermodynamic and dynamical properties of nanosystems with a radical nature at finite temperatures. To highlight some of the present capabilities of TAO-AIMD, we have performed TAO-AIMD simulations to investigate the instantaneous/average radical nature and IR spectra of *n*-acenes (*n* = 2–8) at 300 K. According to the TAO-AIMD simulations, on average, the smaller *n*-acenes (up to *n* = 5) possess a nonradical nature, and the larger *n*-acenes (*n* = 6–8) possess increasing radical nature, showing remarkable similarities to the GS counterparts at 0 K. Besides, the IR spectra of *n*-acenes obtained with the TAO-AIMD simulations are in qualitative agreement with the existing experimental data.

For GS molecules with radical nature [e.g., the larger *n*-acenes (*n* = 6–8)], on average, the radical nature of the molecules can persist in AIMD simulations at finite temperatures. For these molecules, conventional KS-AIMD simulations can therefore be unreliable, and AIMD simulations employing MR electronic structure methods are very likely to be computationally intractable for most molecules. It is thus certainly justified to perform TAO-AIMD simulations for exploring the dynamical information of these molecules.

While only TAO-AIMD (or more specifically, TAO-BOMD) is presented and discussed in this work, it is also possible to combine TAO-DFT with the popular Car-Parrinello MD (CPMD) (Car and Parrinello, 1985) method (i.e., an approximation of the BOMD method) for improved computational efficiency. In addition, it is worth mentioning that a brilliant simulation method combining the advantages of AIMD (for accuracy) and classical MD (for efficiency) has been developed, i.e., the hybrid QM/MM (quantum mechanics/molecular mechanics) method (Warshel and Levitt, 1976), where a small portion (e.g., the reactive portion) of a system is treated with QM and the remaining portion is treated with MM. The QM/MM method has been widely used for the study of very large systems where AIMD simulations are prohibitively expensive (van der Kamp and Mulholland, 2013). To further improve the efficiency of TAO-AIMD, the TAO-DFT-based QM/MM method is thus expected to be useful for the simulations of very large systems (e.g., biomolecules).

While TAO-AIMD is computationally efficient, it could be a very promising approach for the study of the equilibrium thermodynamic and dynamical properties of nanosystems with a radical or non-radical nature at finite temperatures. Nevertheless, a few limitations remain due to a number of assumptions made in TAO-AIMD. For example, the BO approximation is assumed to be valid, and the motion of the nuclei in a system is assumed to evolve only on the GS potential energy surface, wherein non-adiabatic effects are completely ignored. Besides, it is assumed that nuclear quantum effects can be neglected: the nuclear motion is assumed to be governed by the classical equations of motion, rather than the time-dependent nuclear Schrödinger equation. Moreover, it is assumed that TAO-DFT employing the approximate XC and θ-dependent energy functionals (with a system-independent fictitious temperature θ) can provide the exact GS potential energy surface. In addition, similar to KS-AIMD, the real electronic temperature is zero in TAO-AIMD, and it is thus assumed that TAO-AIMD remains applicable for systems at nonzero electronic temperatures. For a system at room temperature, TAO-AIMD should remain applicable (Gaigeot, 2010; Ramírez-Solís et al., 2011; Vitale et al., 2015). However, for a system at a considerably high electronic temperature (e.g., warm dense matter), TAO-AIMD may no longer be applicable (Rüter and Redmer, 2014; Karasiev et al., 2018; Bonitz et al., 2020), wherein AIMD based on the finite-electronic-temperature extension of TAO-DFT will be needed. To lift these limitations, we plan to investigate along some of these lines, and results may be reported elsewhere.

## Data Availability Statement

The raw data supporting the conclusions of this article will be made available by the authors, without undue reservation.

## Author Contributions

J-DC conceived the project. SL performed the calculations. SL and J-DC designed the project, performed the data analysis, and wrote the manuscript. All authors contributed to the article and approved the submitted version.

## Conflict of Interest

The authors declare that the research was conducted in the absence of any commercial or financial relationships that could be construed as a potential conflict of interest.
