# Supplementary material for: TAO-DFT-Based Ab Initio Molecular Dynamics
Source: Front Chem. 2020 Nov 5;8:589432. doi: 10.3389/fchem.2020.589432 (PMC7674663; doi:10.3389/fchem.2020.589432)
Supplement: Supplementary file 1 [file Data_Sheet_2.PDF]

Supplementary Information to: TAO-DFT-Based *Ab Initio* Molecular Dynamics

Shaozhi Li[1] and Jeng-Da Chai[1,2,*]

[1]*Department of Physics, National Taiwan University, Taipei 10617, Taiwan*

[2]*Center for Theoretical Physics and Center for Quantum Science and Engineering, National Taiwan University, Taipei 10617, Taiwan*

[*] Corresponding author. E-mail: jdchai@phys.ntu.edu.tw

LIST OF FIGURES

S1 Time evolution of the difference between the spin-unrestricted and spin-restricted GS electronic energy (i.e., E_0(spin-unrestricted) − E_0(spin-restricted)) of 8-acene, obtained with the TAO-AIMD/KS-AIMD simulations (see Section I in Supplementary Information). 10

S2 Time evolution of the difference between the spin-unrestricted and spin-restricted GS potential energy (i.e., U_0(spin-unrestricted) − U_0(spin-restricted)) of 8-acene, obtained with the TAO-AIMD/KS-AIMD simulations (see Section I in Supplementary Information). 11

S3 Time evolution of the difference between the spin-unrestricted and spin-restricted total energy (i.e., E(spin-unrestricted) − E(spin-restricted)) of 8-acene, obtained with the TAO-AIMD/KS-AIMD simulations (see Section I in Supplementary Information). 11

S4 Time evolution of the symmetrized von Neumann entropy of 2-acene, obtained from four different equilibrated TAO-AIMD trajectories (No.1 to No.4) at 300 K, calculated by TAO-LDA. The TAO-AIMD average and GS values are also shown for comparison. 12

S5 Time evolution of the symmetrized von Neumann entropy of 3-acene, obtained from four different equilibrated TAO-AIMD trajectories (No.1 to No.4) at 300 K, calculated by TAO-LDA. The TAO-AIMD average and GS values are also shown for comparison. 12

S6 Time evolution of the symmetrized von Neumann entropy of 4-acene, obtained from four different equilibrated TAO-AIMD trajectories (No.1 to No.4) at 300 K, calculated by TAO-LDA. The TAO-AIMD average and GS values are also shown for comparison. 13

S7 Time evolution of the symmetrized von Neumann entropy of 5-acene, obtained from four different equilibrated TAO-AIMD trajectories (No.1 to No.4) at 300 K, calculated by TAO-LDA. The TAO-AIMD average and GS values are also shown for comparison. 13

S8 Time evolution of the symmetrized von Neumann entropy of 6-acene, obtained from four different equilibrated TAO-AIMD trajectories (No.1 to No.4) at 300 K, calculated by TAO-LDA. The TAO-AIMD average and GS values are also shown for comparison. . . . 14

S9 Time evolution of the symmetrized von Neumann entropy of 7-acene, obtained from four different equilibrated TAO-AIMD trajectories (No.1 to No.4) at 300 K, calculated by TAO-LDA. The TAO-AIMD average and GS values are also shown for comparison. . . . 14

S10 Time evolution of the active orbital occupation numbers (HOMO−1, HOMO, LUMO, and LUMO+1) of 2-acene, obtained from four different equilibrated TAO-AIMD trajectories (No.1 to No.4) at 300 K, calculated by TAO-LDA. For HOMO/LUMO, the TAO-AIMD average and GS values are also shown for comparison. For brevity, HOMO/LUMO is denoted as H/L. . . . 15

S11 Time evolution of the active orbital occupation numbers (HOMO−1, HOMO, LUMO, and LUMO+1) of 3-acene, obtained from four different equilibrated TAO-AIMD trajectories (No.1 to No.4) at 300 K, calculated by TAO-LDA. For HOMO/LUMO, the TAO-AIMD average and GS values are also shown for comparison. For brevity, HOMO/LUMO is denoted as H/L. . . . 15

S12 Time evolution of the active orbital occupation numbers (HOMO−1, HOMO, LUMO, and LUMO+1) of 4-acene, obtained from four different equilibrated TAO-AIMD trajectories (No.1 to No.4) at 300 K, calculated by TAO-LDA. For HOMO/LUMO, the TAO-AIMD average and GS values are also shown for comparison. For brevity, HOMO/LUMO is denoted as H/L. . . . 16

S13 Time evolution of the active orbital occupation numbers (HOMO−1, HOMO, LUMO, and LUMO+1) of 5-acene, obtained from four different equilibrated TAO-AIMD trajectories (No.1 to No.4) at 300 K, calculated by TAO-LDA. For HOMO/LUMO, the TAO-AIMD average and GS values are also shown for comparison. For brevity, HOMO/LUMO is denoted as H/L. . . . 16

S14 Time evolution of the active orbital occupation numbers (HOMO−1, HOMO, LUMO, and LUMO+1) of 6-acene, obtained from four different equilibrated TAO-AIMD trajectories (No.1 to No.4) at 300 K, calculated by TAO-LDA. For HOMO/LUMO, the TAO-AIMD average and GS values are also shown for comparison. For brevity, HOMO/LUMO is denoted as H/L. 17

S15 Time evolution of the active orbital occupation numbers (HOMO−1, HOMO, LUMO, and LUMO+1) of 7-acene, obtained from four different equilibrated TAO-AIMD trajectories (No.1 to No.4) at 300 K, calculated by TAO-LDA. For HOMO/LUMO, the TAO-AIMD average and GS values are also shown for comparison. For brevity, HOMO/LUMO is denoted as H/L. 17

S16 IR spectra of 2-acene, obtained with the TAO-AIMD simulations at 300 K, calculated by TAO-LDA, where Gaussian window functions with different values of σ (10, 200, 800, and 1600) have been applied (see our paper for details). Experimental (EXP) data [9] are included for comparison. The IR spectra are normalized to have a maximum intensity of one, and for clarity, are vertically offset from each other by the same value. Subfigures show the IR spectra in the 1000–2600 cm^{-1} range. 18

S17 IR spectra of 3-acene, obtained with the TAO-AIMD simulations at 300 K, calculated by TAO-LDA, where Gaussian window functions with different values of σ (10, 200, 800, and 1600) have been applied (see our paper for details). Experimental (EXP) data [9] are included for comparison. The IR spectra are normalized to have a maximum intensity of one, and for clarity, are vertically offset from each other by the same value. Subfigures show the IR spectra in the 1000–2600 cm^{-1} range. 19

S18 IR spectra of 4-acene, obtained with the TAO-AIMD simulations at 300 K, calculated by TAO-LDA, where Gaussian window functions with different values of σ (10, 200, 800, and 1600) have been applied (see our paper for details). Experimental (EXP) data [10–13] are included for comparison. The IR spectra are normalized to have a maximum intensity of one, and for clarity, are vertically offset from each other by the same value. Subfigures show the IR spectra in the 850–2000 cm^{-1} range. 20

S19 IR spectra of 5-acene, obtained with the TAO-AIMD simulations at 300 K, calculated by TAO-LDA, where Gaussian window functions with different values of σ (10, 200, 800, and 1600) have been applied (see our paper for details). Experimental (EXP) data [11–14] are included for comparison. The IR spectra are normalized to have a maximum intensity of one, and for clarity, are vertically offset from each other by the same value. Subfigures show the IR spectra in the 850–2000 cm^{-1} range. 21

S20 IR spectra of 6-acene, obtained with the TAO-AIMD simulations at 300 K, calculated by TAO-LDA, where Gaussian window functions with different values of σ (10, 200, 800, and 1600) have been applied (see our paper for details). The IR spectra are normalized to have a maximum intensity of one, and for clarity, are vertically offset from each other by the same value. Subfigures show the IR spectra in the 1000–2000 cm^{-1} range. 22

S21 IR spectra of 7-acene, obtained with the TAO-AIMD simulations at 300 K, calculated by TAO-LDA, where Gaussian window functions with different values of σ (10, 200, 800, and 1600) have been applied (see our paper for details). The IR spectra are normalized to have a maximum intensity of one, and for clarity, are vertically offset from each other by the same value. Subfigures show the IR spectra in the 1000–2000 cm^{-1} range. 23

S22 IR spectra of 8-acene, obtained with the TAO-AIMD simulations at 300 K, calculated by TAO-LDA, where Gaussian window functions with different values of σ (10, 200, 800, and 1600) have been applied (see our paper for details). The IR spectra are normalized to have a maximum intensity of one, and for clarity, are vertically offset from each other by the same value. Subfigures show the IR spectra in the 1000–2000 cm^{-1} range. 24

LIST OF TABLES

S1 Symmetrized von Neumann entropy of n-acene, obtained with the TAO-AIMD simulations at 300 K and GS calculation, calculated by TAO-LDA. . . . 24

S2 Active orbital occupation numbers (HOMO−1, HOMO, LUMO, and LUMO+1) of n-acene, obtained with the TAO-AIMD simulations at 300 K and GS calculation, calculated by TAO-LDA. For brevity, HOMO/LUMO is denoted as H/L. 25

I. STABILITY OF THE SPIN-UNRESTRICTED AND SPIN-RESTRICTED TAO-AIMD/KS-AIMD SIMULATIONS: PRELIMINARY CALCULATIONS

As mentioned in our paper, because of the constraint of symmetry, the spin-unrestricted and spin-restricted calculations based on an exact electronic structure method should lead to the same energy values for the lowest singlet state (i.e., GS) of n-acene [1–3]. Nevertheless, KS-DFT [4] employing conventional XC energy functionals fail to obey this constraint for the larger n-acenes (which are electronic systems possessing radical nature), leading to the unphysical symmetry-breaking effects in the corresponding spin-unrestricted calculations [5, 6]. In our previous studies [1, 7, 8], the spin-unrestricted and spin-restricted GS (i.e., lowest singlet state) energy values of n-acene (up to $n = 100$), calculated by TAO-LDA (with $\theta = 7$ mhartree) [1], have been found to be essentially the same, leading to essentially no unphysical symmetry-breaking effects in the corresponding spin-unrestricted calculations.

Similarly, starting from prespecified initial nuclear positions and velocities, the spin-unrestricted and spin-restricted AIMD simulations based on an exact electronic structure method, in the NVE ensemble, should yield the same dynamical information, since AIMD is deterministic and there is no unphysical symmetry-breaking problem for the exact electronic structure method. Here, we perform preliminary calculations on 8-acene to examine the possible symmetry-breaking effects in the spin-unrestricted TAO-AIMD/KS-AIMD simulations. In TAO-AIMD simulations, TAO-LDA (i.e., the LDA XC and θ-dependent energy functionals in TAO-DFT) with $\theta = 7$ mhartree is employed. In KS-AIMD simulations, KS-LDA (i.e., the LDA XC energy functional in KS-DFT) is employed.

The initial nuclear positions and velocities of 8-acene are taken from the initial nuclear positions and velocities of 8-acene for an equilibrated TAO-AIMD trajectory (i.e., No.1) at 300 K, calculated by spin-restricted TAO-LDA (as described in the Computational Details of our paper). Subsequently, we perform four separate AIMD simulations, such as the spin-unrestricted and spin-restricted TAO-AIMD/KS-AIMD simulations, in the NVE ensemble, and collect relevant dynamical information (e.g., the GS electronic energy (E_0), GS potential energy (U_0), and total energy (E) at each time t) along each AIMD trajectory for a total of 1,000 time steps ($\approx$ 484 fs).

As shown in Figures S1 to S3, the spin-unrestricted and spin-restricted KS-AIMD simulations can yield distinctly different dynamical information (e.g., distinctly different values

of E_0, U_0, and E at each time t), leading to the unphysical symmetry-breaking effects in the spin-unrestricted KS-AIMD simulations. By contrast, the spin-unrestricted and spin-restricted TAO-AIMD simulations yield essentially the same dynamical information (e.g., essentially the same values of E_0, U_0, and E at each time t), leading to essentially no unphysical symmetry-breaking effects in the spin-unrestricted TAO-AIMD simulations. These are very encouraging results, showing the significance of TAO-AIMD for exploring the dynamical information of large molecules with radical nature.

[1] Chai, J.-D. (2012). Density functional theory with fractional orbital occupations. *J. Chem. Phys.* 136, 154104. doi: 10.1063/1.3703894

[2] Rivero, P., Jiménez-Hoyos, C. A., and Scuseria, G. E. (2013). Entanglement and polyradical character of polycyclic aromatic hydrocarbons predicted by projected Hartree-Fock theory. *J. Phys. Chem. B* 117, 12750–12758. doi: 10.1021/jp401478v

[3] Gryn'ova, G., Coote, M. L., and Corminboeuf, C. (2015). Theory and practice of uncommon molecular electronic configurations. *WIREs Comput. Mol. Sci.* 5, 440–459. doi: 10.1002/wcms.1233

[4] Kohn, W., and Sham, L. J. (1965). Self-consistent equations including exchange and correlation effects. *Phys. Rev.* 140, A1133–A1138. doi: 10.1103/PhysRev.140.A1133

[5] Cohen, A. J., Mori-Sánchez, P., and Yang, W. (2008). Insights into current limitations of density functional theory. *Science* 321, 792–794. doi: 10.1126/science.1158722

[6] Cohen, A. J., Mori-Sánchez, P., and Yang, W. (2012). Challenges for density functional theory. *Chem. Rev.* 112, 289–320. doi: 10.1021/cr200107z

[7] Chai, J.-D. (2014). Thermally-assisted-occupation density functional theory with generalized-gradient approximations. *J. Chem. Phys.* 140, 18A521. doi: 10.1063/1.4867532

[8] Wu, C.-S., and Chai, J.-D. (2015). Electronic properties of zigzag graphene nanoribbons studied by TAO-DFT. *J. Chem. Theory Comput.* 11, 2003–2011. doi: 10.1021/ct500999m

[9] NIST mass spectrometry data center, Wallace, W. E., director, "Infrared Spectra" in NIST chemistry webbook, NIST standard reference database number 69, eds. Linstrom, P. J., and Mallard, W. G., National Institute of Standards and Technology, Gaithersburg MD, 20899, https://doi.org/10.18434/T4D303, (retrieved June 17, 2020).

[10] Hudgins, D. M., and Sandford, S. A. (1998). Infrared spectroscopy of matrix isolated polycyclic aromatic hydrocarbons. 1. PAHs containing two to four rings. *J. Phys. Chem. A* 102, 329–343. doi: 10.1021/jp9834816

[11] Bauschlicher, C. W., Jr., Ricca, A., Boersma, C., and Allamandola, L. J. (2018). The NASA Ames PAH IR spectroscopic database: computational version 3.00 with updated content and the introduction of multiple scaling factors. *ApJS* 234, 32. doi: 10.3847/1538-4365/aaa019

[12] Boersma, C., Bauschlicher, C. W., Jr., Ricca, A., Mattioda, A. L., Cami, J., Peeters, E.,

Sánchez de Armas, F., Puerta Saborido, G., Hudgins, D. M., and Allamandola, L. J. (2014). The NASA Ames PAH IR spectroscopic database version 2.00: updated content, web site, and on(off)line tools. *ApJS* 211, 8. doi: 10.1088/0067-0049/211/1/8

[13] Mattioda, A. L., Hudgins, D. M., Boersma, C., Ricca, A., Peeters, E., Cami, J., Sánchez de Armas, F., Puerta Saborido, G., Bauschlicher, C. W., Jr., and Allamandola, L. J. (2020). The NASA Ames PAH IR spectroscopic database: the laboratory spectra. *ApJS*, submitted.

[14] Hudgins, D. M., and Sandford, S. A. (1998). Infrared spectroscopy of matrix isolated polycyclic aromatic hydrocarbons. 2. PAHs containing five or more rings. *J. Phys. Chem. A* 102, 344–352. doi: 10.1021/jp983482y

FIGURES

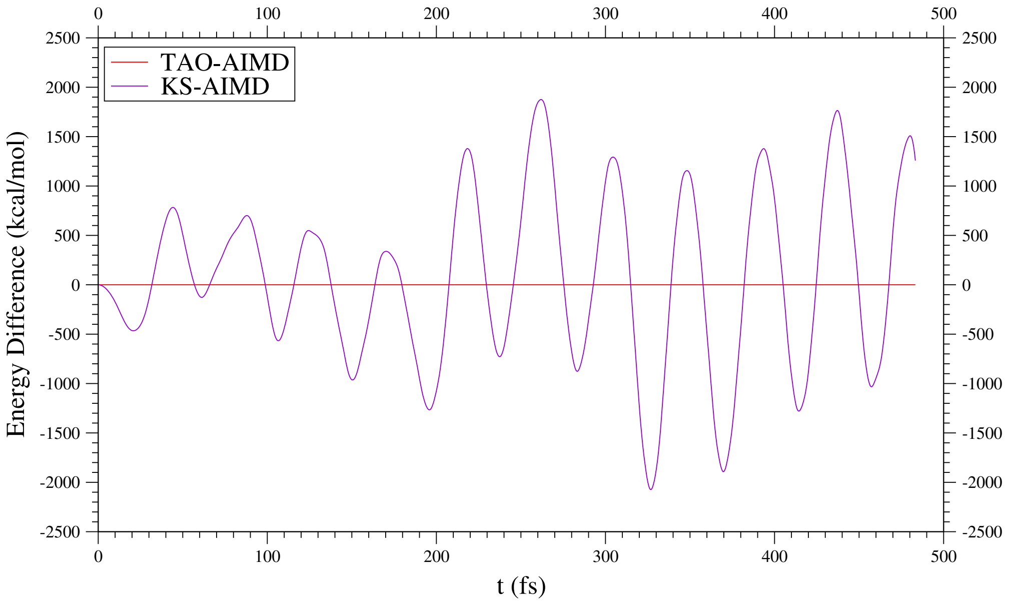

FIG. S1. Time evolution of the difference between the spin-unrestricted and spin-restricted GS electronic energy (i.e., E_0(spin-unrestricted) $-$ E_0(spin-restricted)) of 8-acene, obtained with the TAO-AIMD/KS-AIMD simulations (see Section I in Supplementary Information).

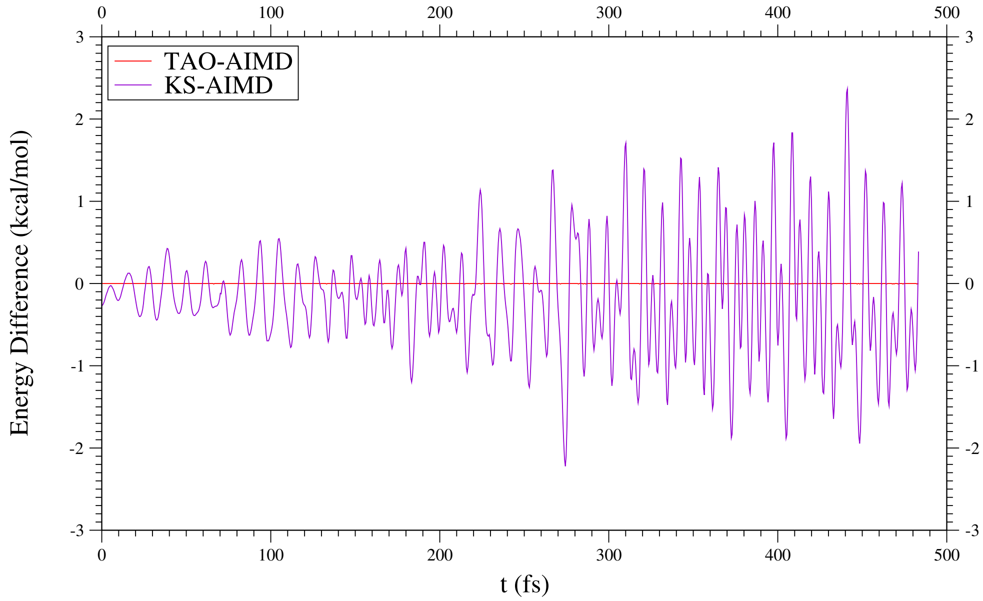

FIG. S2. Time evolution of the difference between the spin-unrestricted and spin-restricted GS potential energy (i.e., U_0(spin-unrestricted) $-$ U_0(spin-restricted)) of 8-acene, obtained with the TAO-AIMD/KS-AIMD simulations (see Section I in Supplementary Information).

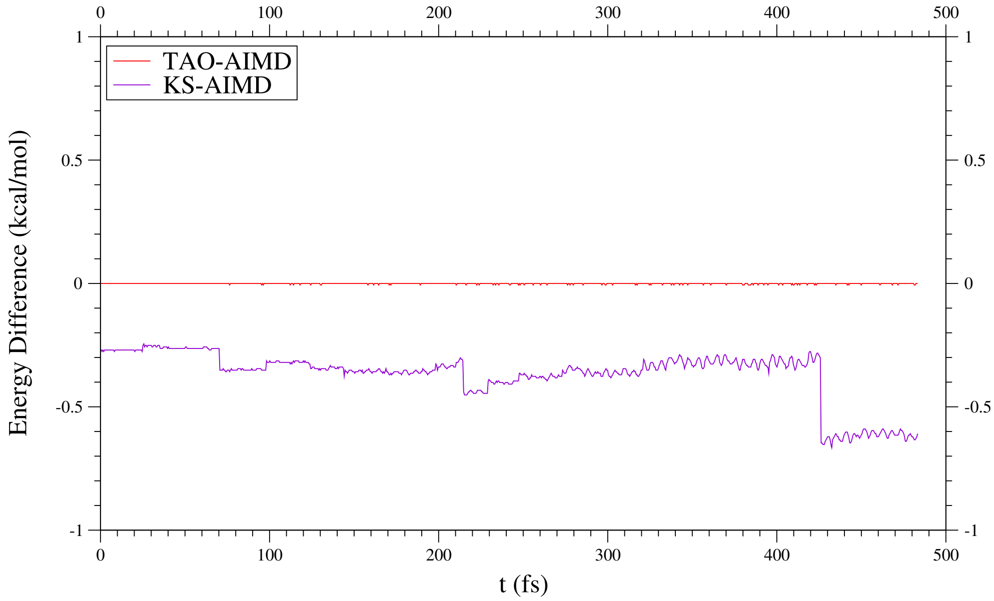

FIG. S3. Time evolution of the difference between the spin-unrestricted and spin-restricted total energy (i.e., E(spin-unrestricted) $-$ E(spin-restricted)) of 8-acene, obtained with the TAO-AIMD/KS-AIMD simulations (see Section I in Supplementary Information).

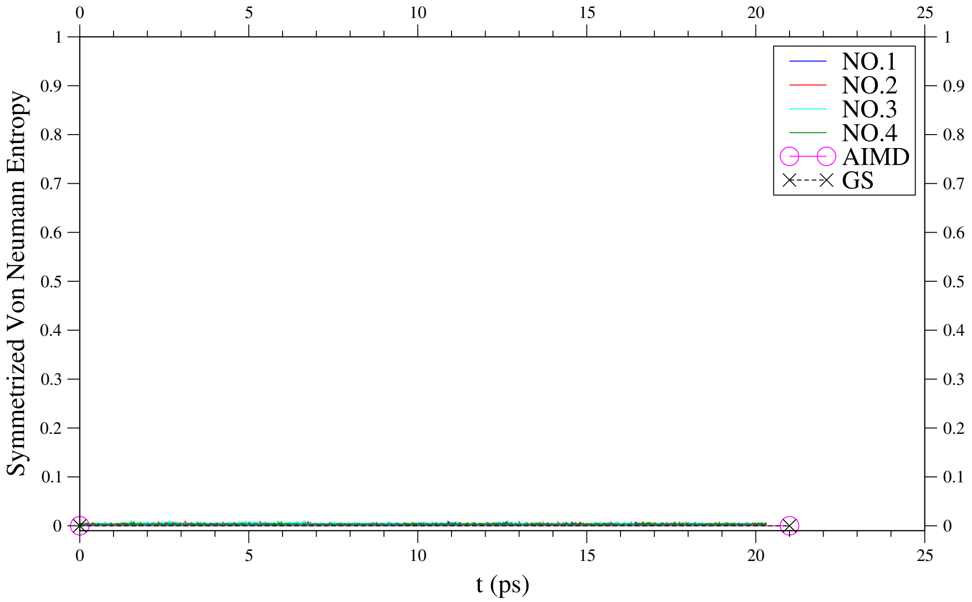

FIG. S4. Time evolution of the symmetrized von Neumann entropy of 2-acene, obtained from four different equilibrated TAO-AIMD trajectories (No.1 to No.4) at 300 K, calculated by TAO-LDA. The TAO-AIMD average and GS values are also shown for comparison.

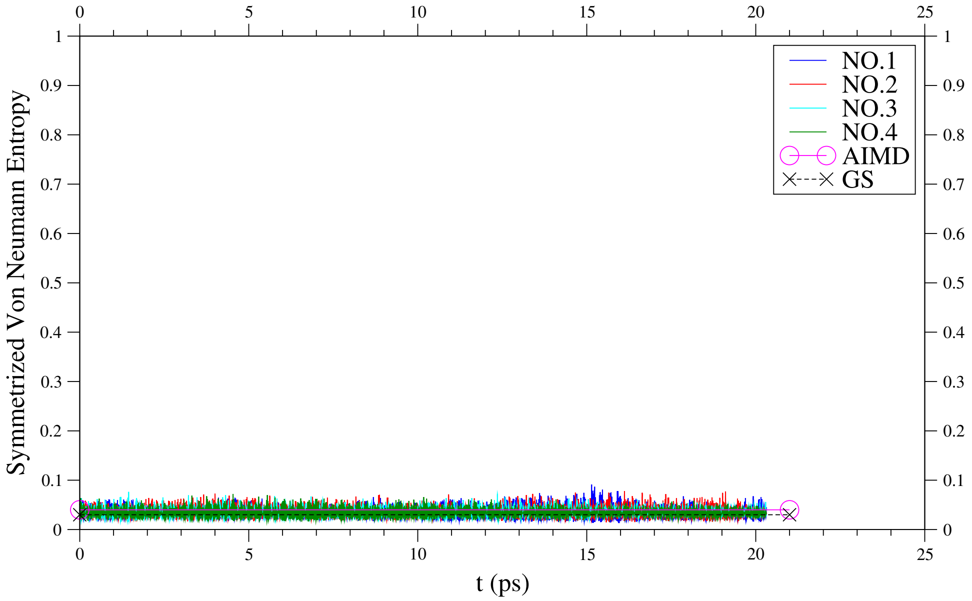

FIG. S5. Time evolution of the symmetrized von Neumann entropy of 3-acene, obtained from four different equilibrated TAO-AIMD trajectories (No.1 to No.4) at 300 K, calculated by TAO-LDA. The TAO-AIMD average and GS values are also shown for comparison.

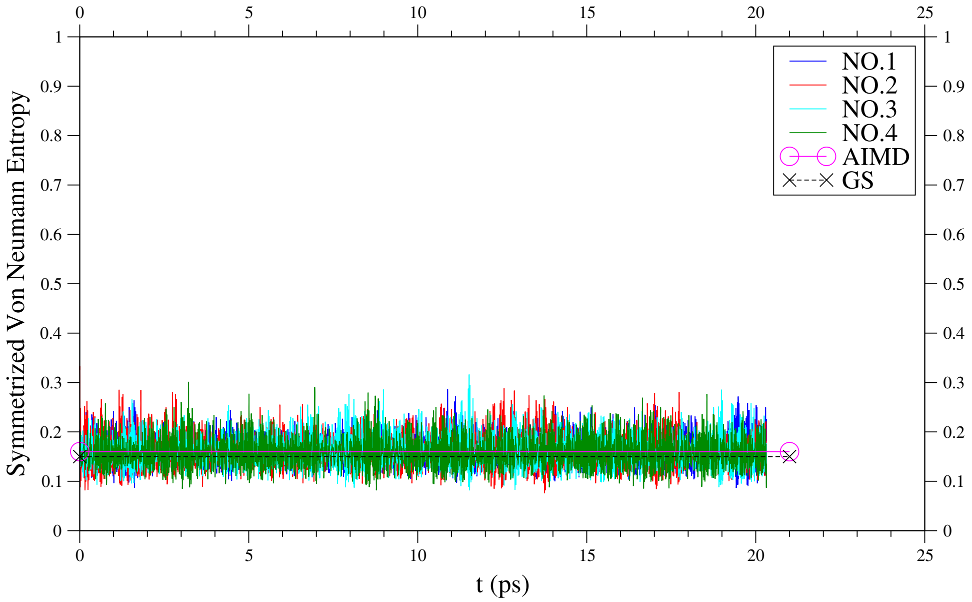

FIG. S6. Time evolution of the symmetrized von Neumann entropy of 4-acene, obtained from four different equilibrated TAO-AIMD trajectories (No.1 to No.4) at 300 K, calculated by TAO-LDA. The TAO-AIMD average and GS values are also shown for comparison.

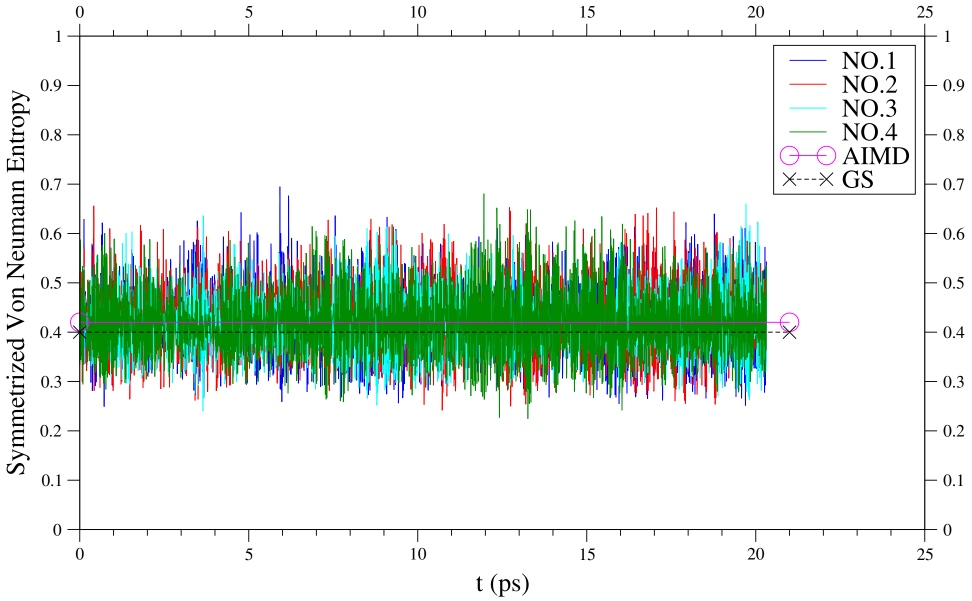

FIG. S7. Time evolution of the symmetrized von Neumann entropy of 5-acene, obtained from four different equilibrated TAO-AIMD trajectories (No.1 to No.4) at 300 K, calculated by TAO-LDA. The TAO-AIMD average and GS values are also shown for comparison.

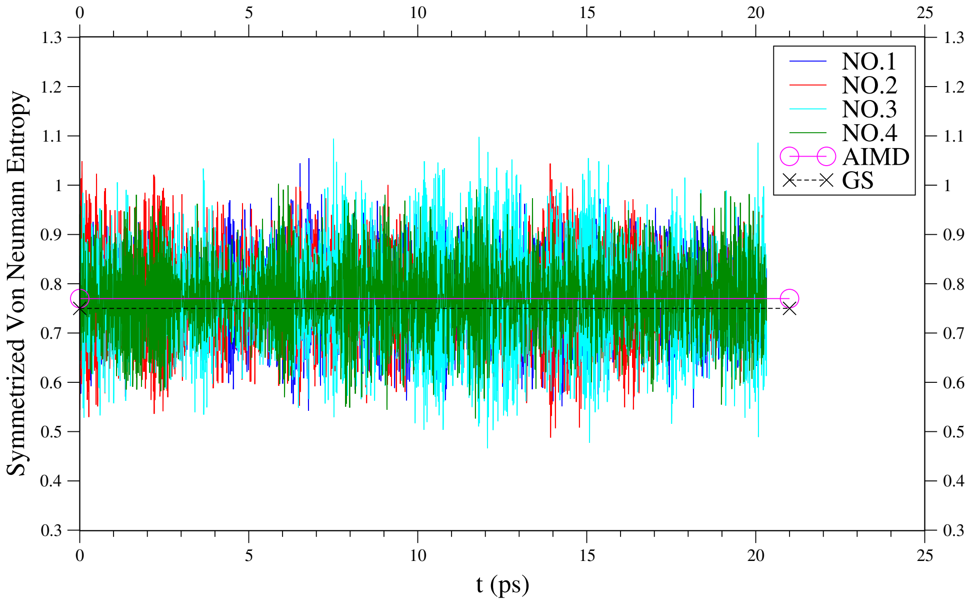

FIG. S8. Time evolution of the symmetrized von Neumann entropy of 6-acene, obtained from four different equilibrated TAO-AIMD trajectories (No.1 to No.4) at 300 K, calculated by TAO-LDA. The TAO-AIMD average and GS values are also shown for comparison.

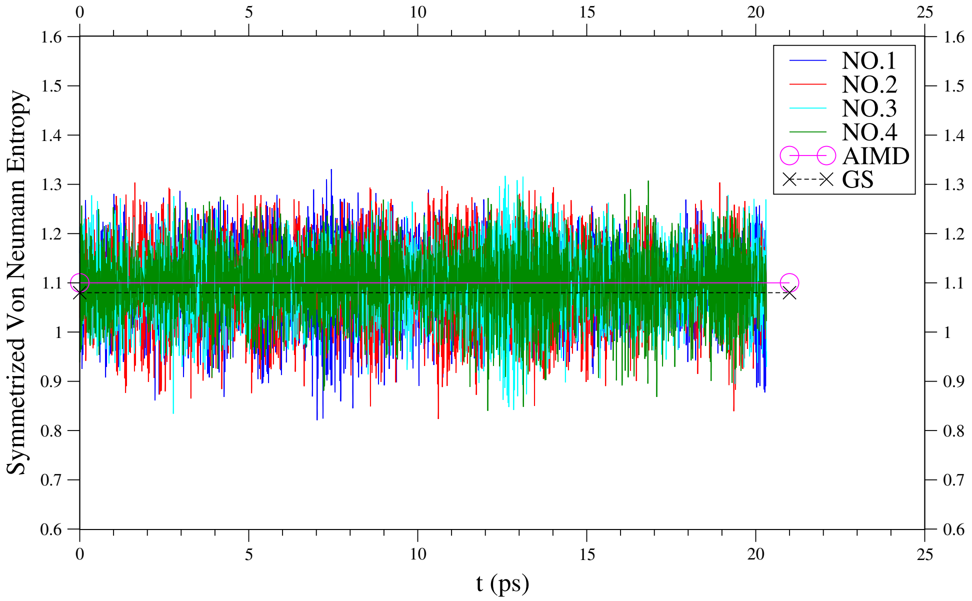

FIG. S9. Time evolution of the symmetrized von Neumann entropy of 7-acene, obtained from four different equilibrated TAO-AIMD trajectories (No.1 to No.4) at 300 K, calculated by TAO-LDA. The TAO-AIMD average and GS values are also shown for comparison.

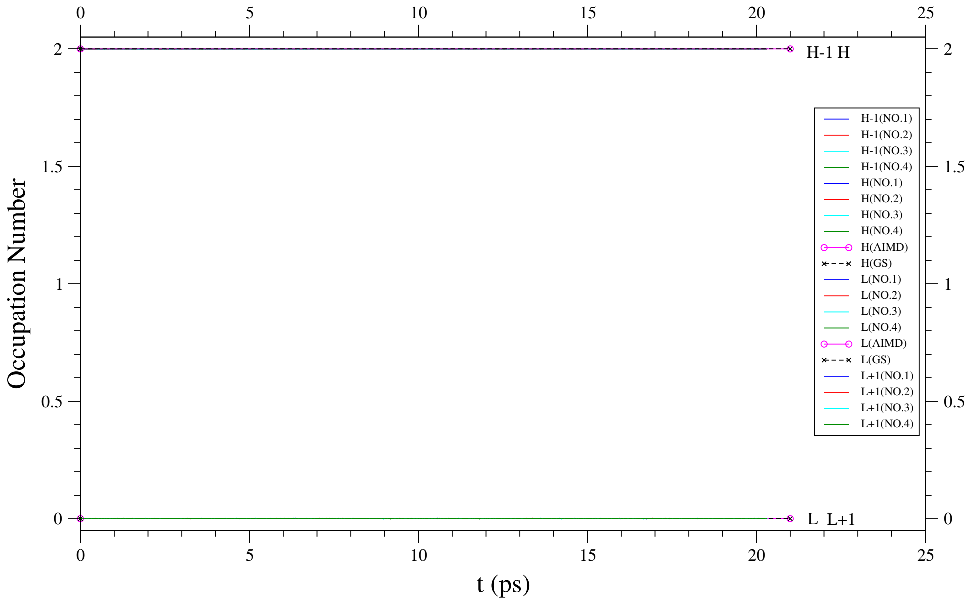

FIG. S10. Time evolution of the active orbital occupation numbers (HOMO−1, HOMO, LUMO, and LUMO+1) of 2-acene, obtained from four different equilibrated TAO-AIMD trajectories (No.1 to No.4) at 300 K, calculated by TAO-LDA. For HOMO/LUMO, the TAO-AIMD average and GS values are also shown for comparison. For brevity, HOMO/LUMO is denoted as H/L.

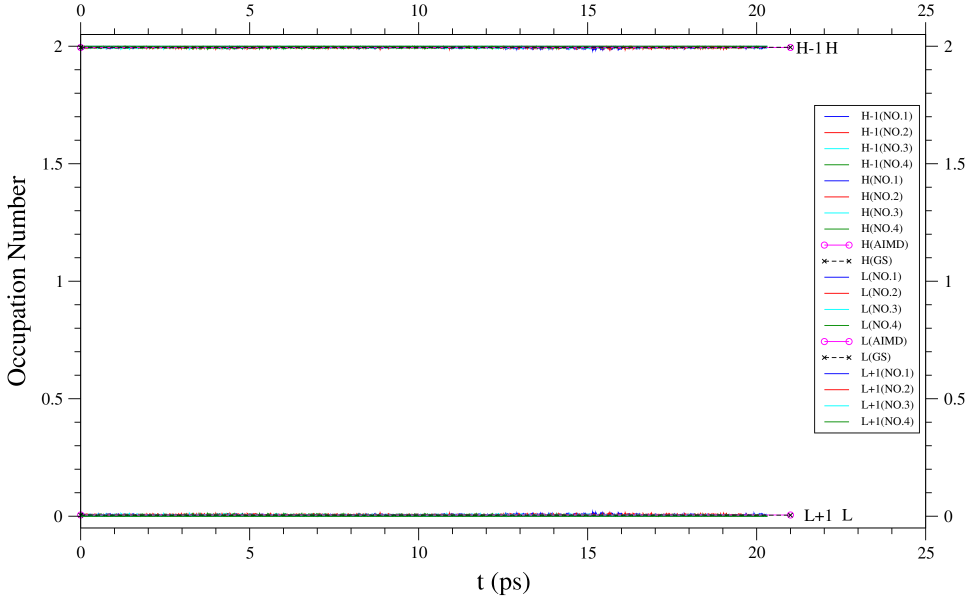

FIG. S11. Time evolution of the active orbital occupation numbers (HOMO−1, HOMO, LUMO, and LUMO+1) of 3-acene, obtained from four different equilibrated TAO-AIMD trajectories (No.1 to No.4) at 300 K, calculated by TAO-LDA. For HOMO/LUMO, the TAO-AIMD average and GS values are also shown for comparison. For brevity, HOMO/LUMO is denoted as H/L.

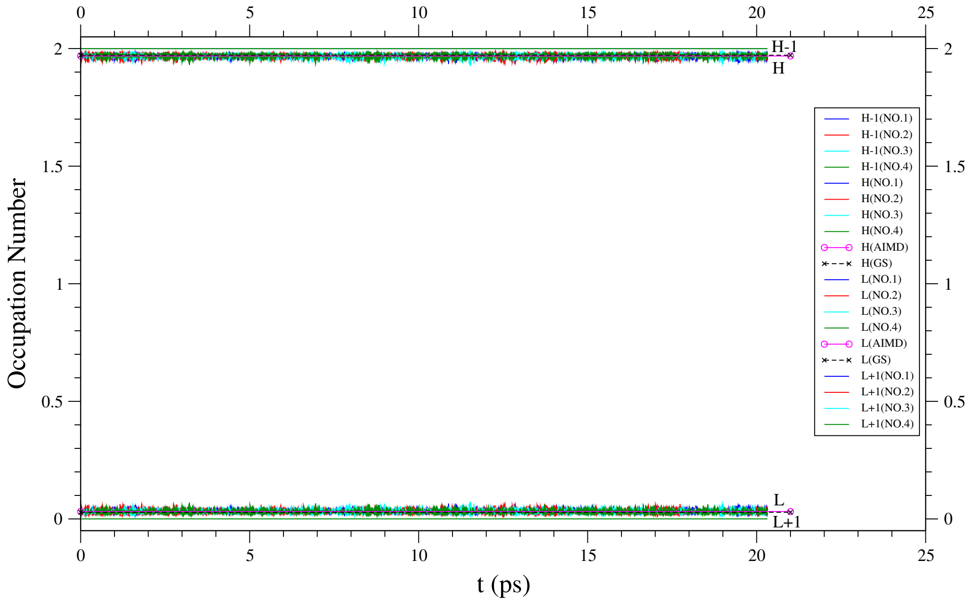

FIG. S12. Time evolution of the active orbital occupation numbers (HOMO−1, HOMO, LUMO, and LUMO+1) of 4-acene, obtained from four different equilibrated TAO-AIMD trajectories (No.1 to No.4) at 300 K, calculated by TAO-LDA. For HOMO/LUMO, the TAO-AIMD average and GS values are also shown for comparison. For brevity, HOMO/LUMO is denoted as H/L.

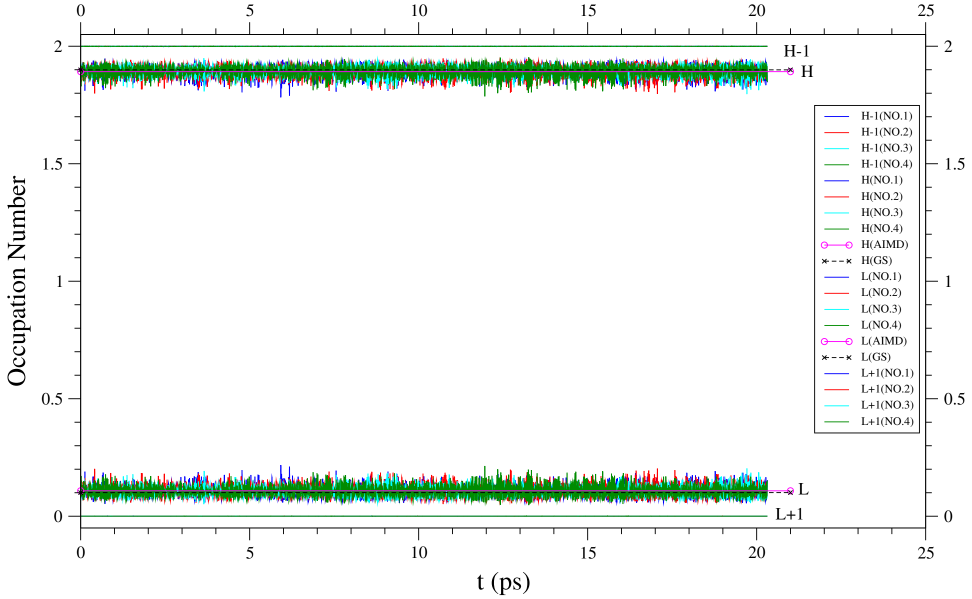

FIG. S13. Time evolution of the active orbital occupation numbers (HOMO−1, HOMO, LUMO, and LUMO+1) of 5-acene, obtained from four different equilibrated TAO-AIMD trajectories (No.1 to No.4) at 300 K, calculated by TAO-LDA. For HOMO/LUMO, the TAO-AIMD average and GS values are also shown for comparison. For brevity, HOMO/LUMO is denoted as H/L.

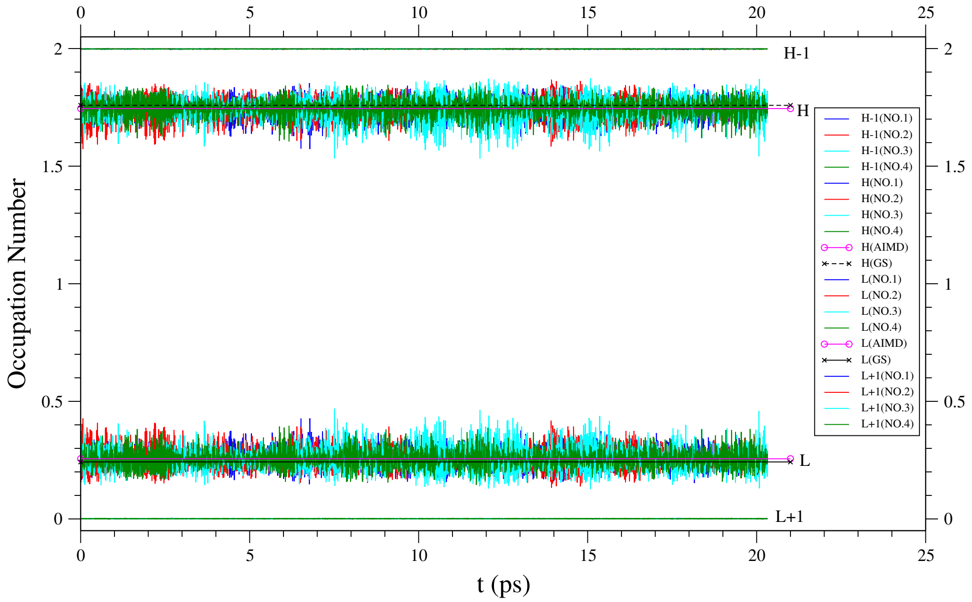

FIG. S14. Time evolution of the active orbital occupation numbers (HOMO−1, HOMO, LUMO, and LUMO+1) of 6-acene, obtained from four different equilibrated TAO-AIMD trajectories (No.1 to No.4) at 300 K, calculated by TAO-LDA. For HOMO/LUMO, the TAO-AIMD average and GS values are also shown for comparison. For brevity, HOMO/LUMO is denoted as H/L.

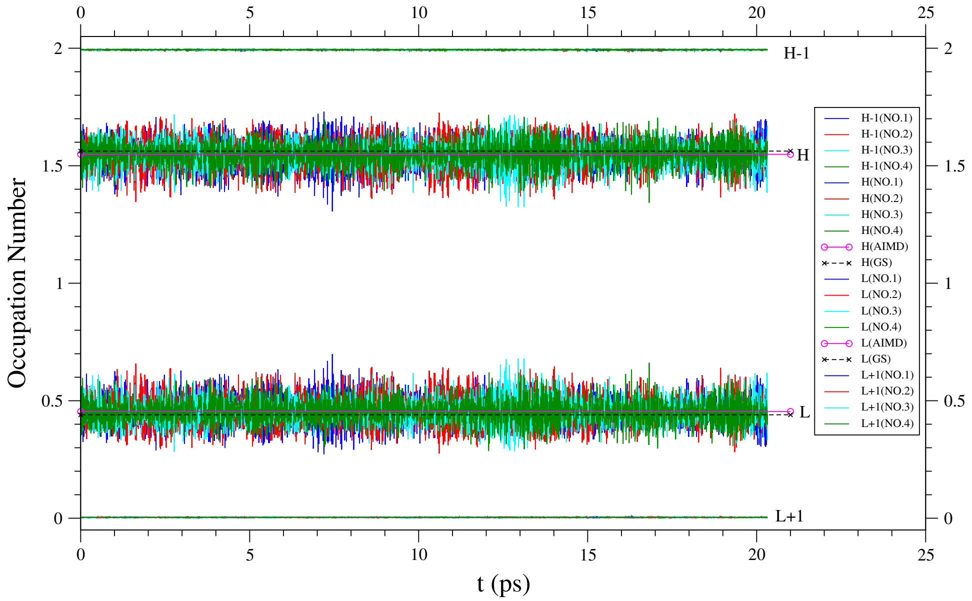

FIG. S15. Time evolution of the active orbital occupation numbers (HOMO−1, HOMO, LUMO, and LUMO+1) of 7-acene, obtained from four different equilibrated TAO-AIMD trajectories (No.1 to No.4) at 300 K, calculated by TAO-LDA. For HOMO/LUMO, the TAO-AIMD average and GS values are also shown for comparison. For brevity, HOMO/LUMO is denoted as H/L.

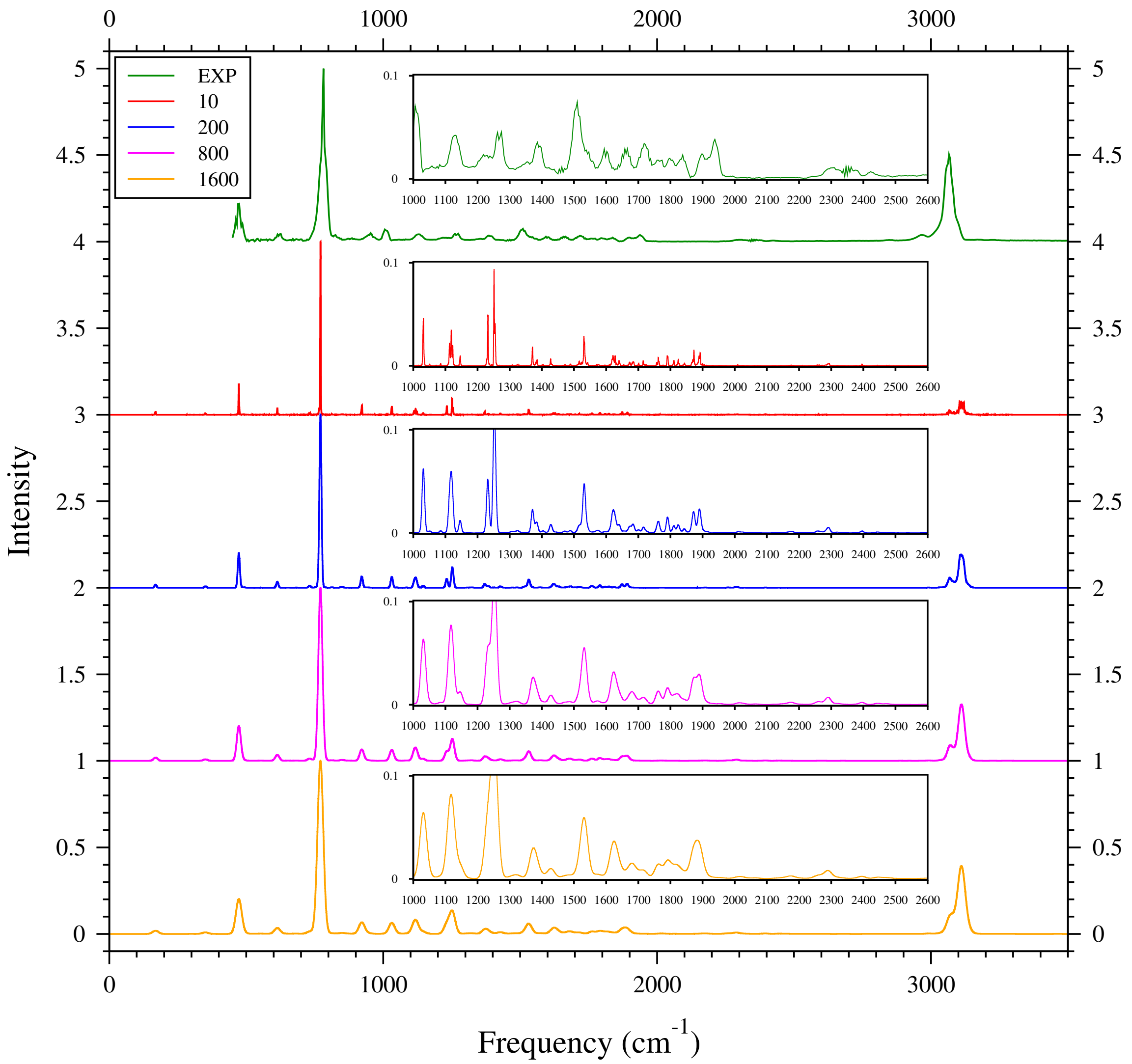

FIG. S16. IR spectra of 2-acene, obtained with the TAO-AIMD simulations at 300 K, calculated by TAO-LDA, where Gaussian window functions with different values of σ (10, 200, 800, and 1600) have been applied (see our paper for details). Experimental (EXP) data [9] are included for comparison. The IR spectra are normalized to have a maximum intensity of one, and for clarity, are vertically offset from each other by the same value. Subfigures show the IR spectra in the 1000–2600 cm^{-1} range.

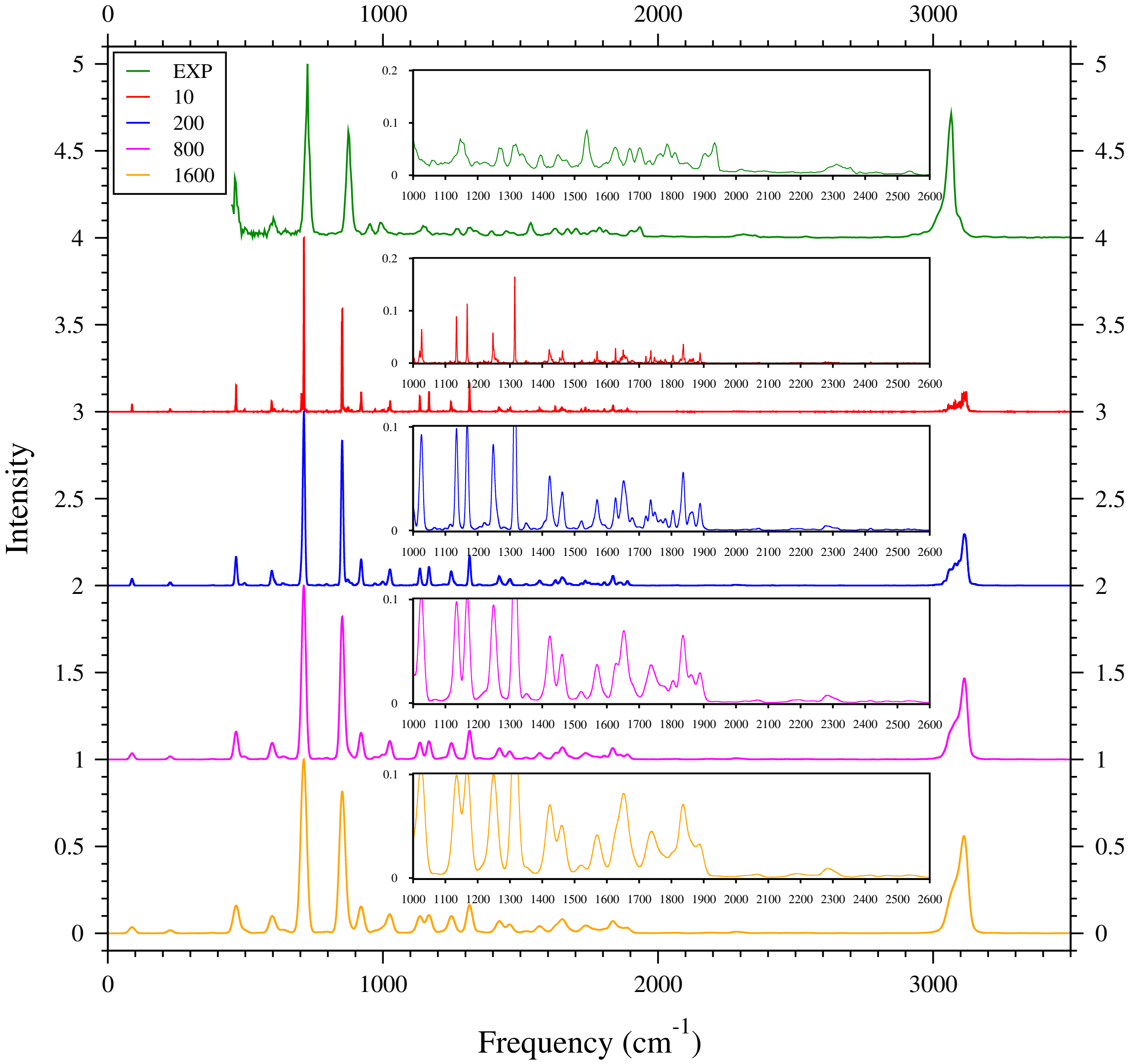

FIG. S17. IR spectra of 3-acene, obtained with the TAO-AIMD simulations at 300 K, calculated by TAO-LDA, where Gaussian window functions with different values of σ (10, 200, 800, and 1600) have been applied (see our paper for details). Experimental (EXP) data [9] are included for comparison. The IR spectra are normalized to have a maximum intensity of one, and for clarity, are vertically offset from each other by the same value. Subfigures show the IR spectra in the 1000–2600 cm^{-1} range.

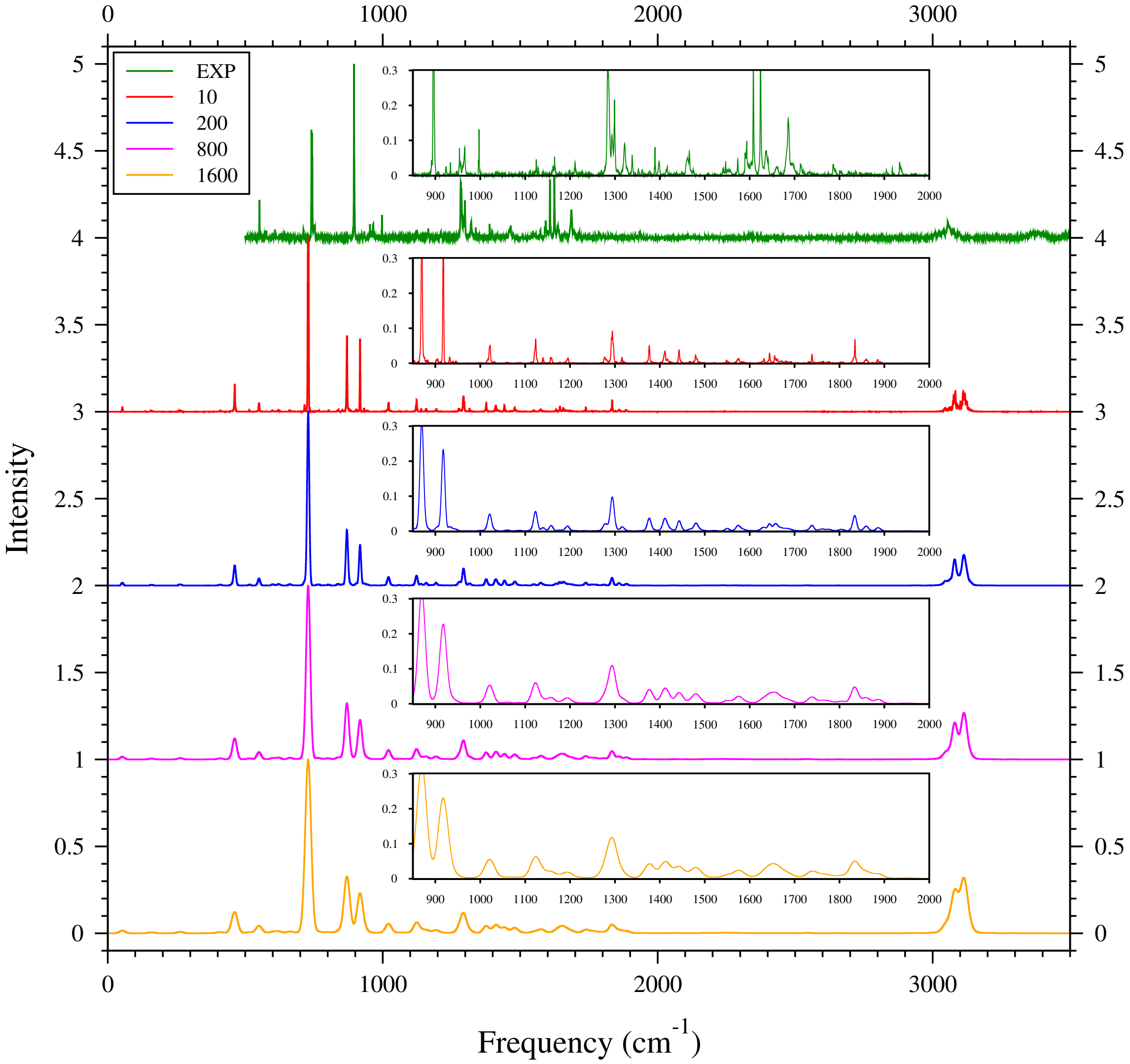

FIG. S18. IR spectra of 4-acene, obtained with the TAO-AIMD simulations at 300 K, calculated by TAO-LDA, where Gaussian window functions with different values of σ (10, 200, 800, and 1600) have been applied (see our paper for details). Experimental (EXP) data [10–13] are included for comparison. The IR spectra are normalized to have a maximum intensity of one, and for clarity, are vertically offset from each other by the same value. Subfigures show the IR spectra in the 850–2000 cm^{-1} range.

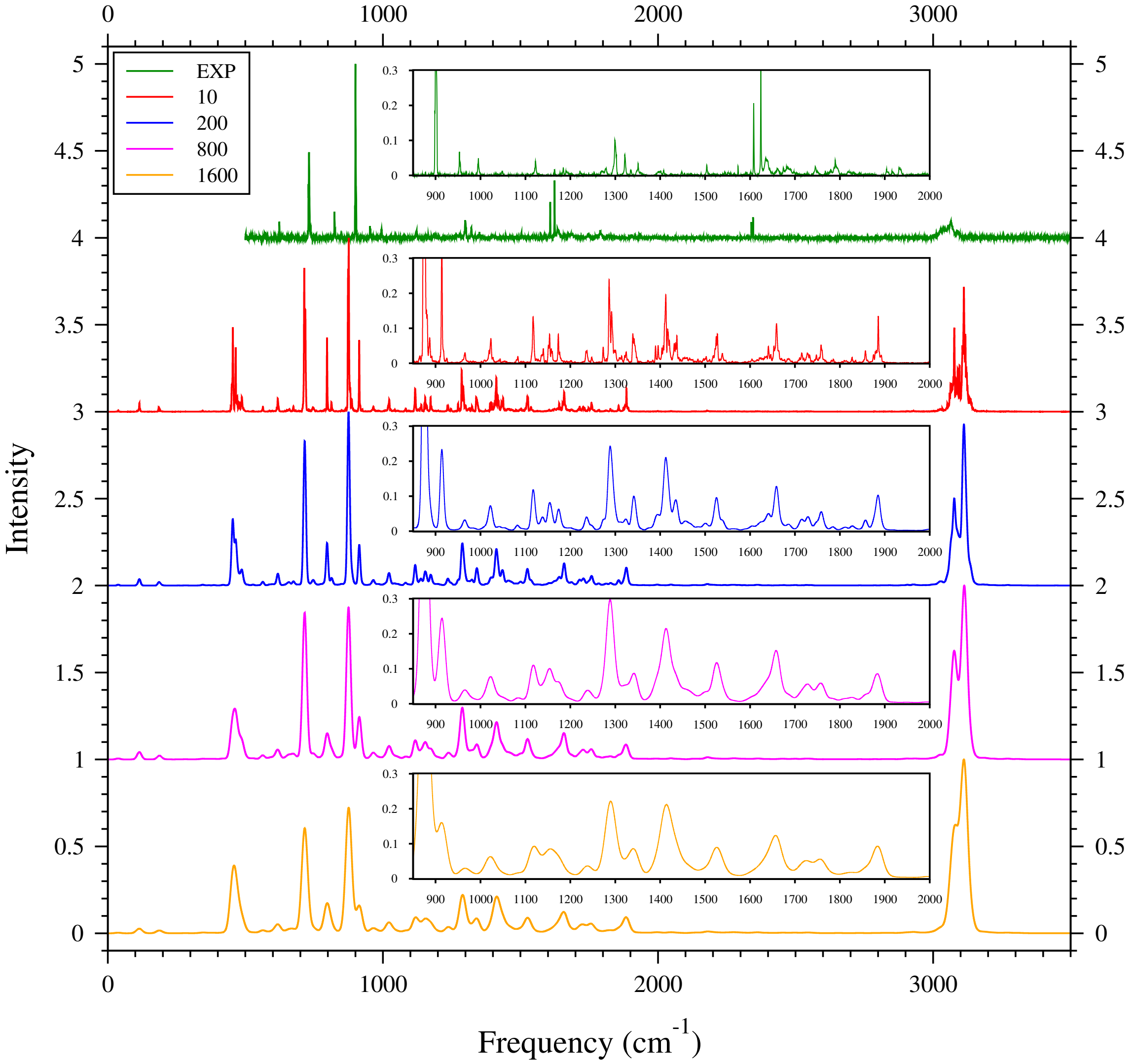

FIG. S19. IR spectra of 5-acene, obtained with the TAO-AIMD simulations at 300 K, calculated by TAO-LDA, where Gaussian window functions with different values of σ (10, 200, 800, and 1600) have been applied (see our paper for details). Experimental (EXP) data [11–14] are included for comparison. The IR spectra are normalized to have a maximum intensity of one, and for clarity, are vertically offset from each other by the same value. Subfigures show the IR spectra in the 850–2000 cm^{-1} range.

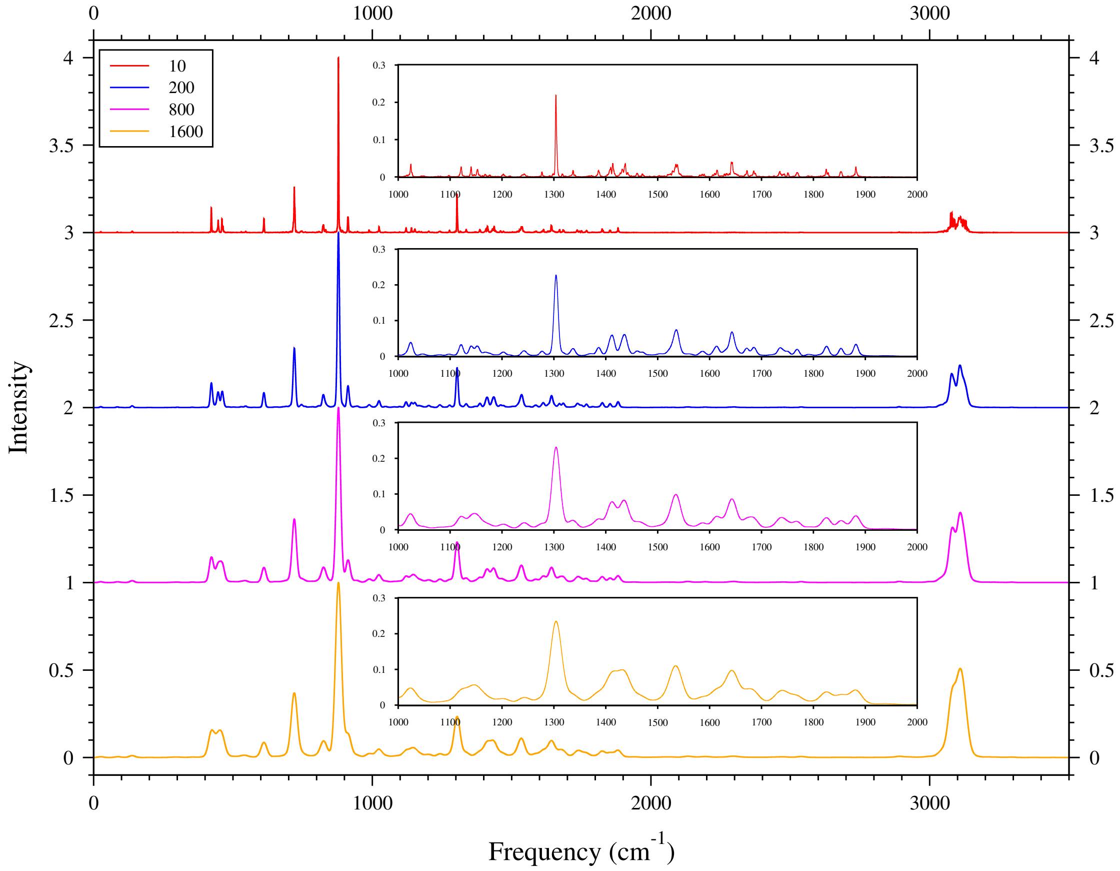

FIG. S20. IR spectra of 6-acene, obtained with the TAO-AIMD simulations at 300 K, calculated by TAO-LDA, where Gaussian window functions with different values of σ (10, 200, 800, and 1600) have been applied (see our paper for details). The IR spectra are normalized to have a maximum intensity of one, and for clarity, are vertically offset from each other by the same value. Subfigures show the IR spectra in the 1000–2000 cm^{-1} range.

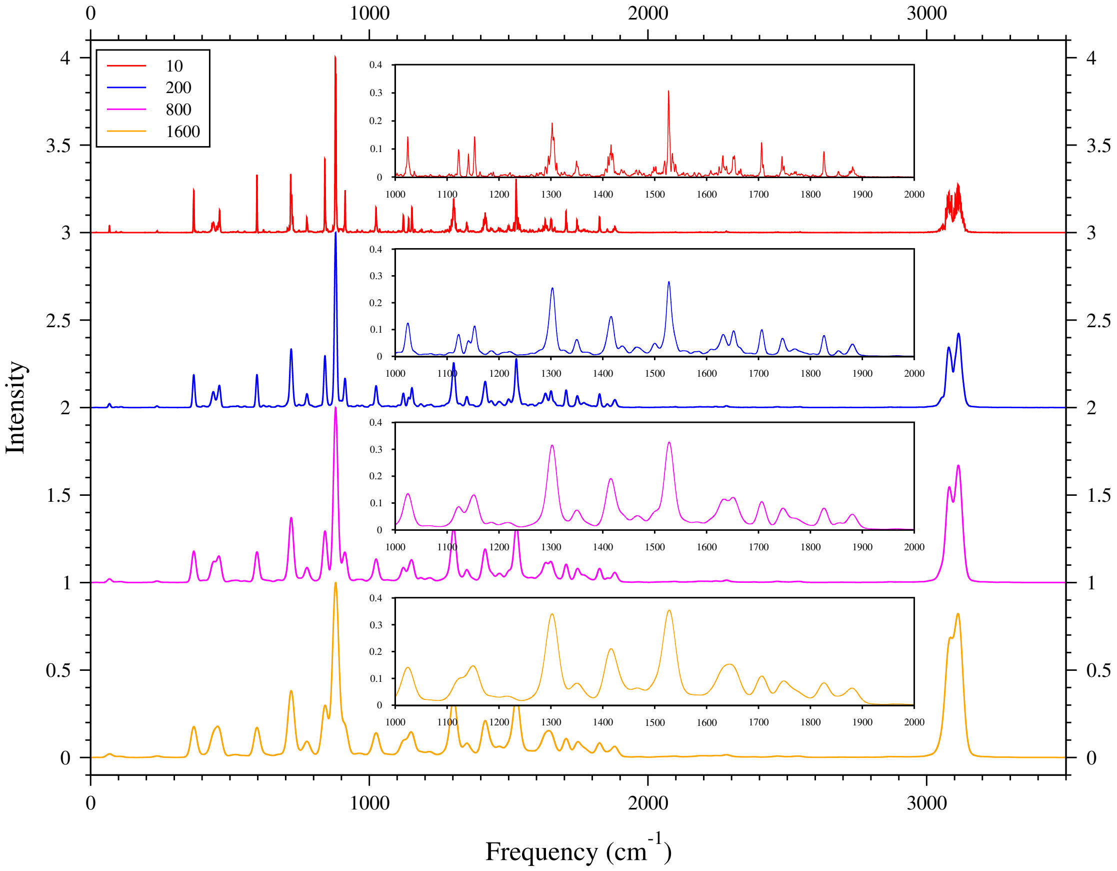

FIG. S21. IR spectra of 7-acene, obtained with the TAO-AIMD simulations at 300 K, calculated by TAO-LDA, where Gaussian window functions with different values of σ (10, 200, 800, and 1600) have been applied (see our paper for details). The IR spectra are normalized to have a maximum intensity of one, and for clarity, are vertically offset from each other by the same value. Subfigures show the IR spectra in the 1000–2000 cm^{-1} range.

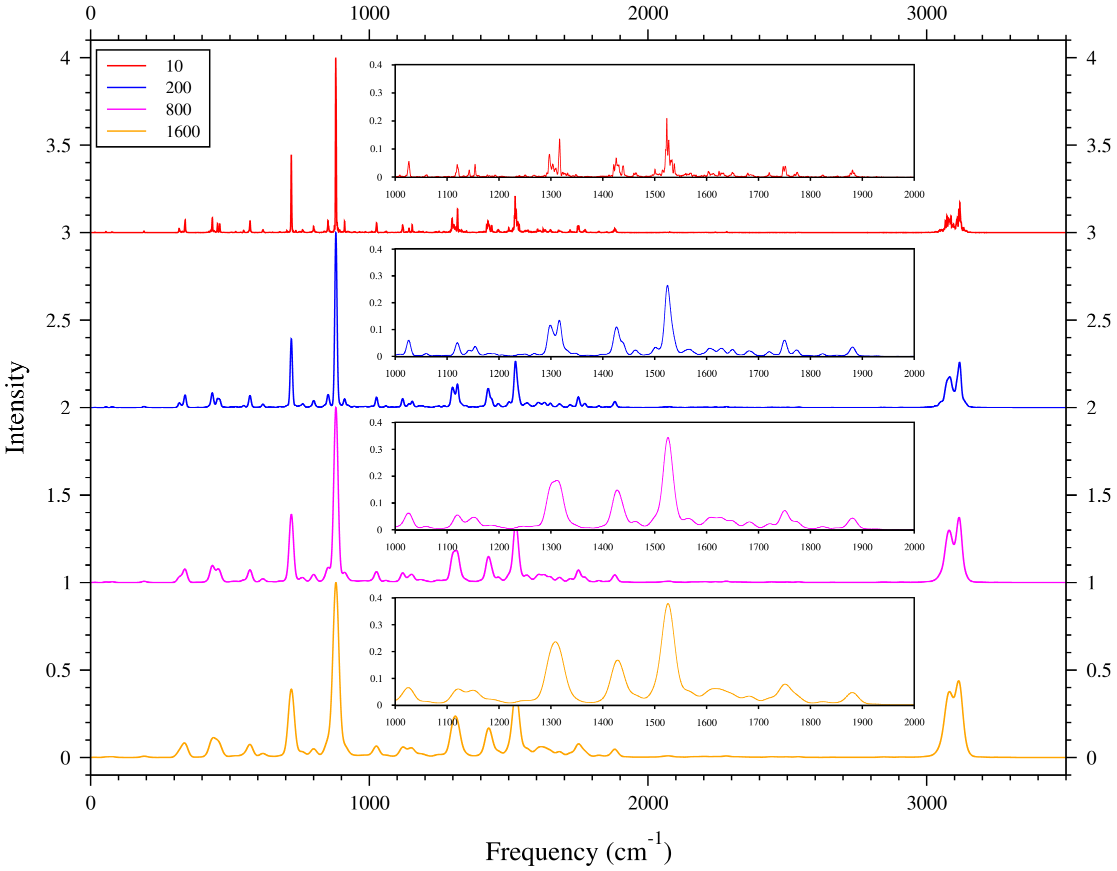

FIG. S22. IR spectra of 8-acene, obtained with the TAO-AIMD simulations at 300 K, calculated by TAO-LDA, where Gaussian window functions with different values of σ (10, 200, 800, and 1600) have been applied (see our paper for details). The IR spectra are normalized to have a maximum intensity of one, and for clarity, are vertically offset from each other by the same value. Subfigures show the IR spectra in the 1000–2000 cm^{-1} range.

TABLES

TABLE S1. Symmetrized von Neumann entropy of n-acene, obtained with the TAO-AIMD simulations at 300 K and GS calculation, calculated by TAO-LDA.

n	AIMD	GS
2	0.00	0.00

3	0.04	0.03
4	0.16	0.15
5	0.42	0.40
6	0.77	0.75
7	1.10	1.08
8	1.35	1.34

TABLE S2. Active orbital occupation numbers (HOMO−1, HOMO, LUMO, and LUMO+1) of n-acene, obtained with the TAO-AIMD simulations at 300 K and GS calculation, calculated by TAO-LDA. For brevity, HOMO/LUMO is denoted as H/L.

	$f_{\text{H-1}}$		f_{H}		f_{L}		$f_{\text{L+1}}$	
n	AIMD	GS	AIMD	GS	AIMD	GS	AIMD	GS
2	2.0000	2.0000	1.9997	1.9998	0.0003	0.0002	0.0000	0.0000
3	2.0000	2.0000	1.9949	1.9957	0.0051	0.0043	0.0000	0.0000
4	2.0000	2.0000	1.9687	1.9719	0.0313	0.0281	0.0000	0.0000
5	1.9997	1.9997	1.8921	1.8997	0.1081	0.1004	0.0001	0.0001
6	1.9983	1.9986	1.7451	1.7587	0.2558	0.2421	0.0009	0.0007
7	1.9938	1.9947	1.5488	1.5624	0.4540	0.4401	0.0034	0.0030
8	1.9830	1.9848	1.3420	1.3529	0.6647	0.6532	0.0105	0.0093
